# Amphiphilic Cu(II) Oxacyclen Complexes: From Oxidative Cleavage to Condensation of DNA

**DOI:** 10.1002/cbic.202500477

**Published:** 2026-01-22

**Authors:** Olga Verbitsky, Sebastián Hinojosa, Amr Mostafa, Deepak Ojha, Ilko Bald, Nora Kulak

**Affiliations:** ^1^ Institute of Chemistry University of Potsdam Karl‐Liebknecht‐Str., 24‐25 14476 Potsdam Germany; ^2^ Institute of Chemistry and Biochemistry Freie Universität Berlin Fabeckstr., 34/36 14195 Berlin Germany

**Keywords:** Cu(II) complexes, DNA binding, DNA cleavage, DNA condensation, reactive oxygen species

## Abstract

Cu(II) complexes with monoalkylated oxacyclen ligands (C_12_, C_16_, and C_18_) have been investigated regarding their interaction with DNA by different methods: circular dichroism, UV/VIS (ultraviolet‐visible) and fluorescence spectroscopy as well as by gel electrophoresis. The results demonstrate that the complexes can cleave DNA through both hydrolytic and oxidative mechanisms, with hydroxyl radicals and hydrogen peroxide identified as the reactive oxygen species involved. The targeted incorporation of alkyl chains significantly enhances the DNA‐binding affinity of the Cu(II) complexes, and the length of the alkyl substituents plays an important role, as they can interact with the major groove of the DNA. Alkylation is the determining structural factor responsible for the enhanced DNA interaction, since such an interaction is not observed with unsubstituted complexes. Moreover, the length of the alkyl chains significantly influences this behavior, as longer substituents induce a concentration‐dependent DNA aggregation, a phenomenon absent in the nonalkylated analog. This aggregation and condensation behavior is examined using atomic force microscopy and dynamic light scattering. Moreover, DNA/small molecule interactions are also investigated using molecular dynamics simulations.

## Graphical abstract

Monoalkylated Cu(II) oxacyclen complexes show a dual DNA effect: shorter alkyl chains enable ROS‐mediated DNA cleavage, whereas longer chains cause strong DNA condensation/aggregation. Thus, chain length controls activity. Using circular dichroism, UV/visible and fluorescence spectroscopy, atomic force microscopy, dynamic light scattering, and molecular dynamics, we demonstrate that amphiphilic Cu(II) complexes are promising DNA modulators.

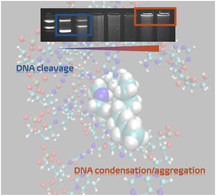

## 1 Introduction

Deoxyribonucleic acid (DNA) is the carrier of genetic information within living cells.^[^ ^1^ ^]^ The central dogma of molecular biology, formulated by Francis Crick in 1958, describes the directional flow of genetic information toward proteins in living organisms,^[^ ^2^ ^]^ known as transcription and translation.^[^ ^3^ ^]^ Normally, the DNA sequence is highly stable under physiological conditions. Nevertheless, mutations, environmental factors and epigenetic modifications can impair the function of DNA and lead to damage and mutations. These changes are a major contributor to the development of cancer.^[^ ^4^ ^,^ ^5^ ^]^

In 2022, around 9.74 million people worldwide died from cancer, based on estimates by the International Agency for Research on Cancer (IARC).^[^ ^6^ ^]^ The effective treatment of cancer remains a significant challenge in modern oncology. Metal‐based chemotherapeutic agents, including cisplatin, oxaliplatin, and carboplatin, interact through covalent bonds with the purine bases in DNA by forming intra‐ and inter‐strand crosslinking, thus interrupting the cell replication and growth of tumor cells through induction of apoptosis (programed cell death).^[^ ^7^, ^8^ ^–^ ^9^ ^]^ Despite the great success of platinum‐based chemotherapeutics such as cisplatin, there are still considerable limitations, including the development of resistance, high systemic toxicity and side effects such as neurotoxicity, ototoxicity, and nausea. These limitations have led to the search for alternative anticancer drugs that can provide greater efficacy and tolerability. In particular, complexes based on endogenous transition metals such as copper, zinc, cobalt, and iron have shown promising results. Moreover, these endogenous metals lead to an estimated lower systemic toxicity in comparison to platinum‐based chemotherapeutic agents.^[^ ^10^ ^]^

In recent years, copper‐based complexes have broadly been investigated for therapeutic applications.^[^ ^11^ ^]^ Copper plays a central role in biological processes, such as oxygen transport and cell signaling.^[^ ^12^ ^]^ In addition, it shows a higher selectivity for tumor cells,^[^ ^13^ ^]^ as these often have an increased copper metabolism, which makes copper promising for cancer research. Copper‐ and platinum‐based drugs differ fundamentally in their mechanisms of action. Whereas platinum complexes exert their cytotoxicity mainly through DNA adduct formation and subsequent inhibition of replication, copper‐based agents may function as metallonucleases by promoting the generation of reactive oxygen species (ROS). This oxidative stress induced by ROS leads to oxidative DNA damage when high enough, which consequently causes apoptosis in cancer cells.^[^ ^14^, ^15^ ^–^ ^16^ ^]^ The structure and accessibility of DNA are critical factors determining its susceptibility to ROS making DNA condensation an important step in the process.^[^ ^17^ ^]^ DNA condensation is a natural process that contributes to genome organization, particularly through the formation of chromatin structures. During cell division, pronounced chromatin condensation leads to a temporary halt in both transcription and DNA replication, which directly affects cell proliferation.^[^ ^18^ ^]^ Furthermore, DNA condensation plays a key role along with DNA fragmentation in apoptosis. Particularly noteworthy is that cationic copper(II) complexes have been reported to induce reversible DNA condensation and trigger apoptosis in tumor cells, such as MG‐63 cell line.^[^ ^19^ ^]^ Other metal‐based systems have demonstrated similar behavior: dinuclear Cu(II) complexes with polybenzimidazole ligands exhibit potent condensing ability and hold promise as nonviral gene vectors. This insight opens up new therapeutic approaches, suggesting that targeted DNA condensation could be used for tumor treatment. It demonstrates that DNA condensation is not only a fundamental mechanism of genome organization, but also a promising target for the development of innovative cancer therapies.^[^ ^20^ ^]^ Such controlled interactions are highly dependent on the design of the ligand. In Cu(II) complexes, chelating ligands stabilize the metal center while also modulate DNA‐binding properties.^[^ ^21^, ^22^ ^–^ ^23^ ^]^ Among the various ligand systems that have investigated, macrocyclic polyamines such as cyclen (1,4,7,10‐tetraazacyclododecane or[12]aneN4) have received particular attention. First synthesized in 1961 by Hermann Stetter,^[^ ^24^ ^]^ cyclen has been extensively studied due to its outstanding ability to coordinate a variety of metal ions, such as Cu(II).^[^ ^25^ ^]^ Current studies have focused on the further derivatization of the Cu(II)‐cyclen complex, such as via N‐functionalization, to increase DNA binding affinity and thus DNA cleavage activity.^[^ ^22^ ^,^ ^23^ ^]^ In order to achieve an impact on donor characteristics of the ligand and redox properties of the resulting complex, we have investigated Cu(II) cyclen complexes, where the coordinating N atoms were substituted by O and S atoms.^[^ ^26^ ^,^ ^27^ ^]^ The studies consistently show that heteroatom substituents significantly influence the DNA cleavage activity (O > S > N). The stabilization of both the Cu(I) and Cu(II) states by different donor atoms is of central importance for the efficiency of oxidative DNA cleavage involving ROS. This is due to the ability to provide balanced stability in both redox states while allowing effective reactivity with ROS. In addition to influencing the redox properties, the introduction of hydrophobic alkyl chains into metal complexes is an important strategy for increasing their biological activity, especially for interactions with biomolecular targets such as DNA and proteins, as well as membrane permeability and cellular uptake. Metallosurfactants, amphiphilic metal complexes of hydrophilic and lipophilic moieties, have been shown to self‐assemble and form aggregates in solution, penetrate biological membrane and bind selectively to hydrophobic regions of proteins.^[^ ^28^ ^]^ There has been no report, however, on aggregation phenomena of DNA when such amphiphilic metal complexes have been used. Furthermore, the effects of alkyl substituents of varying lengths in Cu(II) cyclen on the cleavage of proteins were investigated. Monoalkylated Cu(II) complexes exhibited up to a 100‐fold increase in cleavage activity toward bovine serum albumin (BSA) compared to unsubstituted analogs. Longer alkyl chains led to higher proteolytic activity, as they improved the interaction of the complexes with hydrophobic regions of the target proteins.^[^ ^29^ ^,^ ^30^ ^]^

**Figure 1:**
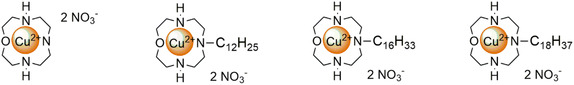
Structural representation of **CuL0**, **CuL1**, **CuL2**, and **CuL3** complexes.

In the present study, we systematically investigated a series of monoalkylated Cu(II) oxacyclen complexes with different alkyl chain lengths (C_12_, C_16_, and C_18_) to examine how both the heteroatom substitution (N → O) in combination alkyl chain derivatization affects their interaction with DNA. The structures of the investigated complexes are shown in **Figure** **1**.

## 2 Results and Discussion

### 2.1 DNA Cleavage Studies: Hydrolytic versus Oxidative Cleavage

Plasmid DNA is very convenient as a substrate for studying DNA cleavage ability of the complexes for its potential to adopt an open‐circular conformation.^[^ ^31^ ^]^ The capability of the complexes to induce DNA cleavage was evaluated by converting the supercoiled plasmid DNA (form I) to the open‐circular/nicked (form II) or linear (form III) DNA form by means of agarose gel electrophoresis.^[^ ^32^ ^]^ Based on their mechanism of action, Cu(II) complexes can cleave DNA in different ways: either by oxidative cleavage or by a hydrolytic cleavage pathway. For oxidative cleavage to take place, the presence of a redox active metal (e.g., Cu(II)) in a metal complex is required. The first step involves the activation of the metal ion through a suitable reducing agent such as ascorbate (ascH^−^).^[^ ^33^ ^,^ ^34^ ^]^

The concentration‐dependent DNA cleavage activity of the complexes **CuL1**, **CuL2,** and **CuL3** (3–50 µM) toward pBR322 plasmid DNA was investigated in the presence of ascorbate (0.32 mM) as reducing agent. To imitate physiological conditions in vitro, MOPS buffer with a pH of 7.4 was used.

**Figure 2:**
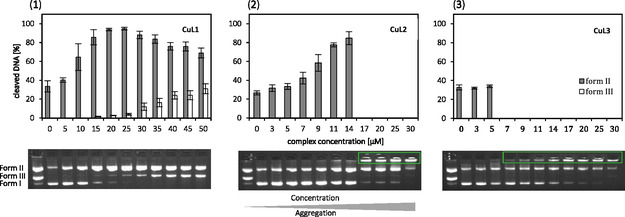
Comparison of the cleavage activity of 1) **CuL1**, 2) **CuL2,** and 3) **CuL3** (in µM) regarding pBR322‐Plasmid‐DNA (0.025 µg µL^−1^) in MOPS buffer (50 mM, pH7.4) in the presence of ascorbic acid (0.32 mM) after 2 h of incubation at 37 °C. Visualization of a representative agarose gel (**bottom).** Quantitative evaluation as mean value from three measurements with the standard deviation as error bar (**top**).

Generally, all complexes showed approximately equivalent DNA cleavage activity at a concentration of ≤10 µM. Compound **CuL1** with the shortest alkyl chain (C_12_), almost completely converted DNA from form I into DNA form II at a complex concentration of 20 µM. Additionally, ≈3% of DNA form III was generated. The DNA is increasingly cleaved into the linear form III (35% at 50 µM) with increasing **CuL1** concentration (**Figure 2**.1). When reaching 70 µM of **CuL1** the bands in the gel exhibit blurriness and block the electrophoretic migration of the DNA fragments, indicating the formation of DNA‐complexes aggregates (Figure S1.2, Supporting Information). In the case of **CuL2**, a DNA‐complex adduct forms progressively as the concentration of the complexes increases: at concentrations below 14 µM, **CuL2** cleaves the DNA into form II (90%), whereas at concentrations ≥17 µM, the DNA exist in a strongly compacted form remaining in the gel loading wells (Figure 2.2.). Extending the chain length from C_16_ to C_18_ in **CuL3** leads to aggregation already at 7 µM as indicated by green frames in Figure 2.3, making the evaluation of DNA cleavage more difficult. Therefore, no definitive statements can be made regarding the DNA cleavage at higher complex concentrations. The resulting DNA adducts assumingly adopt a bulky shape which, owing to their size, can only partially migrate within the gel. As a result, they partly remain trapped within the gel pockets. One possible explanation for this behavior could be the interaction of the alkyl chain with the DNA grooves, leading to the formation of the aggregates. It can be concluded that as the alkyl chain length of the complex increases, the fewer complex molecules are needed to initiate DNA condensation.

A similar phenomenon was observed in earlier work by us. It was found that multiply 9,10‐anthraquinone (AQ)‐substituted Cu(II)‐cyclen complexes induce the formation of DNA aggregates at micromolar concentrations. This was triggered by the intercalation of the AQ moieties into DNA duplexes, which facilitated aggregation process.^[^ ^35^ ^]^ The results obtained in the present work are consistent with these findings. Regarding the mechanism for aggregation, however, we assume some differences: whereas AQ is a typical intercalator, alkyl chains are not.

To investigate the potential interaction of complex **CuL1** (C_12_ chain, 40 µM) at the major groove of DNA, a cleavage experiment was performed using methyl green (MG) as major groove binder and thus inhibitor of the cleavage reaction.^[^ ^17^ ^,^ ^36^ ^]^ As Figure S1.5, Supporting Information, shows, the presence of MG resulted in an inhibition of the nuclease activity of **CuL1**. This clearly indicates major groove binding of **CuL1**, and it can be assumed that this is the case also for the other similar complexes **CuL2** and **CuL3**. Additionally, the binding of **CuL1** to the major groove of CT‐DNA can be investigated using a DNA groove‐binding assay. In this experiment, **CuL1** is titrated into a solution of MG at pH 7.4, and the interaction is analyzed by fluorescence measurements, as shown in Figure S1.6, Supporting Information. MG binds reversibly to CT‐DNA and forms a stable MG–DNA complex at neutral pH with a fluorescence peak at 672 nm upon excitation at 633 nm.^[^ ^37^ ^]^ The fluorescence intensity of the solution decreases with increasing **CuL1** concentration, indicating that **CuL1** competes with MG for binding within the major groove of CT‐DNA.

Potential nuclease activity of the ligands was studied under identical conditions, exemplarily for **L2** (C_16_ chain). As expected in the absence of the redox active metal ion Cu^2+^, ligand **L2** did neither exhibit cleavage activity nor DNA aggregation (Figure S1.7., Supporting Information). DNA aggregation induced by **CuL1–CuL3** can thus clearly be attributed to the combination of the Cu^2+^ ion and the ligand. Accordingly, the nonalkylated Cu(II) oxacyclen **CuL0** demonstrated the ability to cleave DNA, although no aggregation was observed even at the concentration as high as 320 µM.^[^ ^38^ ^]^ Additionally, the effect of the buffer on the cleavage of plasmid DNA was investigated by replicating the experiment using **CuL3** with HEPES (2‐(4‐(2‐hydroxyethyl)‐1‐piperazineethanesulfonic acid) as the buffer. HEPES, similar to MOPS, is commonly used in analytical and biological studies due to its inability to competitively coordinate with Cu(II) ions.^[^ ^39^ ^]^ No significant difference in the DNA cleavage activity was observed when compared to experiments performed in MOPS buffer (Figure S1.8, Supporting Information). An influence of the buffer on the DNA cleavage and aggregation activities by Cu(II) complexes can thus be ruled out.

In the following, cleavage of pBR322 plasmid DNA by Cu(II) complexes **CuL1–CuL3** was performed under hydrolytic conditions, with incubation of 24 h and in the absence of external reducing agent (Figure S2.1–S2.3, Supporting Information). The general mechanism of hydrolysis happens by nucleophilic attack of the phosphate backbone of DNA by water or hydroxide ions, forming a five‐coordinate phosphate and subsequent cleaving leading to strand breaking.^[^ ^14^, ^15^ ^–^ ^16^ ^,^ ^40^ ^]^ **CuL1** exhibits no DNA cleavage activity under the applied conditions. However, in the case of **CuL2** and **CuL3** the linear DNA form III was detected in addition to DNA form II, even at a concentration of 3 µM complex in the sample. Moreover, aggregation of the DNA with **CuL2** and **CuL3** was observed at concentrations ≥9 µM of the complexes, which then does not allow quantification of DNA cleavage at this concentration or higher.

**Figure 3:**
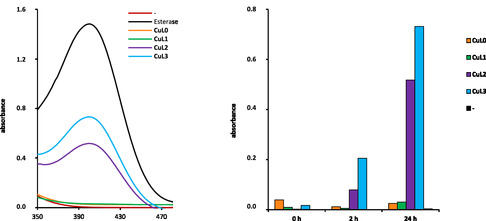
Absorption spectra of BNPP (0.08 mM) in MOPS buffer (50 mM, pH 7,4) (**red line**) in the presence of phosphodiesterase I enzyme (0.05 U mL^−1^) (**black line**) or **CuL0**–**CuL3** (0.2 mM) after 24 h incubation by 37 °C (**left**). Absorption maxima of BNPP showing the intensity corresponding to *p*‐nitrophenol production by **CuL0–CuL3** (0.2 mM) in MOPS buffer (50 mM, pH 7.4) after 0, 2, and 24 h of incubation at 37 °C (**right**).

Hydrolytic DNA cleavage can be assessed using methods such as the phosphate ester bis(4‐nitrophenyl) phosphate (BNPP) assay or the religation assay.^[^ ^40^ ^]^ In the religation assay, the DNA form II generated by the complexes is incubated with DNA ligase and then analyzed by agarose gel electrophoresis. Hydrolytic activity can be demonstrated by the reappearance of DNA form I, which indicates successful religation of hydrolytically cleaved DNA ends. However, since Cu(II) complexes showed very low hydrolytic activity, and aggregation was observed at higher concentrations, the religation assay is not suitable for comparing the hydrolysis DNA cleavage activity in the present study. Therefore, the hydrolytic activity of **CuL0–CuL3** was evaluated using the active phosphate ester bis(4‐nitrophenyl)phosphate (BNPP) as a model substrate, which mimics the phosphodiester backbone of DNA.^[^ ^40^ ^,^ ^41^ ^]^ The complexes **CuL0–CuL3** (0.2 mM) were incubated with BNPP (0.08 mM) in MOPS (50 mM) for 0, 2, and 24 h. The results after 24 h incubation of the Cu(II) complexes are shown in **Figure 3** (spectra for incubation times of 0 and 2 h in Figure S3, Supporting Information). The positive control was tested using enzymatic cleavage by phosphodiesterase I (0.05 U mL^−1^). The cleavage was analyzed by means of a UV/VIS (ultraviolet‐visible) experiment utilizing the characteristic absorption bands of BNPP at 290 nm^[^ ^41^ ^]^ and monitoring of the formation of the hydrolysis product *p*‐nitrophenol (NP) at 400 nm. Consistent with a prior investigation, it was confirmed that the nonalkylated Cu(II) complex **CuL0** was hydrolytically inactive^[^ ^38^ ^]^ (Figure 3 orange line). After 24 h incubation of BNPP with complex **CuL1** containing the shortest alkyl chain (C_12_)**,** no change in the absorption band at 400 nm was observed. **CuL2** and **CuL3** exhibited the ability to cleave BNPP within a 2 h the incubation period. Extending the incubation time to 24 h resulted in an increased conversion of the BNPP substrate by **CuL2** and **CuL3**, which was reflected in a fivefold increase in the intensity of the absorption band at 400 nm. Therefore, the hydrolysis pathway emerges as a conceivable mechanism for DNA cleavage induced by **CuL2** and **CuL3**, which the C_18_ complex demonstrated highest activity. This dependance on the alkyl chain length suggests a supramolecular aggregation phenomenon like micelle formation.^[^ ^28^, ^29^ ^–^ ^30^ ^]^

Accordingly, König et al. observed a positive impact on the hydrolytic cleavage activity by means of the formation of micelles and vesicles from amphiphilic Zn(II) cyclen complexes, which was also investigated using the BNPP assay. The observed increase in reaction rates was attributed to the high local concentration of coordinated Zn(II) ions within the metallomicelles.^[^ ^42^ ^]^ The high hydrolysis rates observed with BNPP suggest that, in the case of **CuL2** and **CuL3**, micelle formation due to the localization of multiple complex units in close proximity leads to increased hydrolytic cleavage of the phosphate backbone.

**Figure 4:**
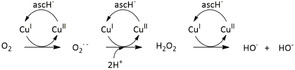
Schematic mechanism of ROS generation through oxygen reduction and Cu(II)/Cu(I) redox cycling in the presence of ascorbate.

Generally, hydrolytic and oxidative DNA cleavage by artificial metallonucleases occurs catalytically. Hydrolytic cleavage by metal ions can, for example, be facilitated by activation of the phosphate ester due to the Lewis acidity of the metal ions.^[^ ^43^ ^]^ Whereas in the oxidative cleavage process, the metal complexes are activated by a reducing agent. Consequently, a Cu(II)/Cu(I) redox cycle can be generated.^[^ ^44^ ^]^ In the form of Cu(I), it can activate molecular O_2_. In a sequence of reactions, ROS result as products. The main ROS involved in this mechanism are hydroxyl radicals (^•^OH), singlet oxygen (^1^O_2_), superoxide radical anions (O_2_^**.**−^), and hydrogen peroxide (H_2_O_2_) based on this mechanism.^[^ ^33^ ^,^ ^34^ ^]^ (**Figure 4**). The generated ROS are unable to diffuse far from their point of origin before they react nonselectively, which means that ROS‐generating compounds with high DNA affinity might be more efficient DNA cleavers. ROS can extract hydrogen atoms from the deoxyribose or oxidize the nucleobases, leading thus to unstable sites within the DNA or inducing DNA damage.^[^ ^33^, ^34^, ^35^, ^36^, ^37^, ^38^, ^39^, ^40^, ^41^, ^42^, ^43^ ^–^ ^44^ ^]^

### 2.2 ROS Detection Assay

#### 2.2.1 Determination by Gel Electrophoresis

To demonstrate that the cleavage activity of the Cu(II) complexes follows an oxidative mechanism, ROS quenching experiments were performed. Representatively, **CuL1** (20 µM) was incubated with pBR322 plasmid DNA (0.025 µg µL^−1^) in the presence of various ROS scavengers for 2 h at 37 °C. The quench assay for **CuL2** and the **CuL3** could not be conducted due to aggregation at the required complex concentrations. The following quenchers were used: DMSO (200 mM) for hydroxyl radical (^•^OH), NaN_3_ (10 mM) for singlet oxygen (^1^O_2_), pyruvate (2.5 mM) for hydrogen peroxide (H_2_O_2_), and SOD (625 U mL^−1^) for superoxide radical anions (O_2_^**.**−^).^[^ ^45^ ^,^ ^46^ ^]^ If ROS are involved in cleavage, then DNA cleavage should be inhibited or reduced in the presence of the corresponding ROS scavenger compared to the cleavage activity without a ROS scavenger.^[^ ^42^ ^]^

**Figure 5:**
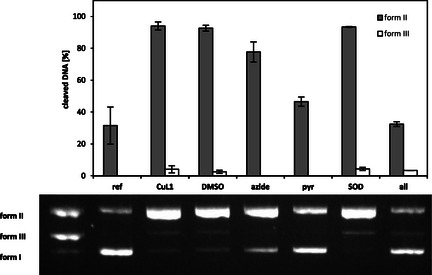
Cleavage activity of the complex **CuL1** (20 µM) toward pBR322 plasmid DNA (0.025 µg µL^−1^) in MOPS buffer (50 mM, pH 7.4) in the presence of ascorbate (0.32 mM) and corresponding ROS scavengers. Incubation for 2 h at 37 °C. **Lane 1**: DNA ladder (form I, form II, form III); **lane 2**: DNA reference; **lane 3**: **CuL1** (20 µM): **lane 4**: **CuL1** + DMSO (200 mM); **lane 5**: **CuL1** +NaN_3_ (10 mM); **lane 6**: **CuL1** + pyruvate (2.5 mM); **lane 7: CuL1** + SOD (625 U mL^−1^); **lane 8: CuL1** + all ROS scavenges. Visualization of a representative agarose gel (**bottom**). Quantitative evaluation as mean value from three measurements with the standard deviation as error bar (**top**).

As shown for **CuL1** in **Figure** **5**, the addition of DMSO does not lead to an inhibition within uncertainties on the DNA cleavage. In the presence of NaN_3_ and pyruvate, the cleavage activity is reduced. This result suggests that only singlet oxygen (^1^O_2_) and hydrogen peroxide (H_2_O_2_) are involved in the oxidative DNA cleavage process of **CuL1**.

#### 2.2.2 Detection by Fluorescence Using Terephthalate (TPA) and Pentafluorobenzenesulfonyl Fluorescein (PBSF)

Additionally, ROS involved in oxidative cleavage were detected using a fluorescence assay. Specifically, fluorogenic dyes such as terepthalate (TPA) and pentafluorobenzenesulfonyl fluorescein (PBSF) were used for the selective and reproducible detection of hydroxyl radicals (^•^OH) and hydrogen peroxide (H_2_O_2_), respectively.^[^ ^47^ ^]^

The detection of hydrogen peroxide generated by Cu(II) complexes was achieved through perhydrolysis of PBSF at near‐physiological pH. Upon reaction with H_2_O_2_, the sulfonate was selectively cleaved, resulting in a strong fluorescence emission band at 513 nm when excited at a wavelength of 485 nm^[^ ^47^ ^]^ (Figure S4.1., Supporting Information). The complexes **CuL1–CuL3**, in the presence of ascorbic acid and PBSF, exhibit fluorescence intensity at ≈513 nm, increasing in the following order **CuL1** < **CuL2** < **CuL3** (Figure S4.2.–3, Supporting Information). The reaction of the fluorogen with the generated ROS, quenched by pyruvate as a ROS scavenger, exhibits a correspondingly reduced fluorescence intensity, indicating the cleavage (perhydrolysis) of PBSF to fluorescein. For the nonalkylated **CuL0**, no significant fluorescence is observed compared to the intrinsic fluorescence of PBSF, indicating that **CuL0** generally does not exhibit a notable generation of H_2_O_2_. Due to the increased production of H_2_O_2_ associated with the increasing chain length of alkyl substituents in the complexes, it is assumed that oxidative DNA cleavage, involving ROS, becomes more pronounced with longer chain length. **CuL3** is presumed to be the most efficient in this regard.

Nonfluorescent TPA reacts selectively with hydroxyl radicals through a hydroxycyclohexadienyl radical intermediate to produce the highly fluorescent 2‐hydroxyterephthalate (HTPA), exhibiting an emission maximum at 428 nm.^[^ ^47^ ^,^ ^48^ ^]^

The negative control, TPA only under physiological conditions, is not fluorescent, whereas the fluorescence intensity for the conditions where **CuL0–CuL3** (40 mM) and a reducing agent are present increased strongly (Figure S4.4 and S4.5, Supporting Information). The addition of DMSO (200 mM) as a quencher within the reaction system resulted in a significant decrease in fluorescence intensity, indicating the ability of all Cu(II) complexes to generate hydroxyl radicals (^•^OH) in the presence of a reducing agent. The generation of ^•^OH was detected for the complexes, although it was slightly lower than for unalkylated **CuL0**. This is a remarkable result, as the detection of ROS by gel electrophoresis has only a minimal effect on the ^•^OH‐induced DNA cleavage reaction mediated by **CuL2**. A possible explanation for the discrepancy between the TPA assay and the gel electrophoresis quench assay is the extremely short half‐life (on range of nanoseconds) and the limited diffusion range of ^•^OH.^[^ ^48^ ^]^ This means that ^•^OH radicals react quickly with DNA at the site of their formation. When ^•^OH are generated in close proximity to the DNA, such as through DNA‐bound Cu(II) complexes, they react immediately with the DNA before they can diffuse away or be scavenged by radical inhibitor DMSO. This assumption is supported by the results of the ROS quench assay, in which no significant inhibition of DNA cleavage was observed in the presence of DMSO. This suggests that ^•^OH radicals are generated in close proximity to the plasmid DNA, such that an effective scavenging reaction by DMSO is not able due to their short lifetime and limited diffusion range of the ^•^OH radicals (see Figure 4, Lane 4). In contrast, in the TPA assay, the ^•^OH radicals are generated in the complete reaction solution, allowing DMSO to neutralize them efficiently, resulting in a strong inhibition of fluorescence. Furthermore, differences in the concentrations of the complex (20 vs. 40 µM) could influence the formation of ROS. It is conceivable that at lower complex concentrations, the formation of hydroxyl radicals does not occur, as previously formed hydrogen peroxide is completely consumed by DNA cleavage, but the ^•^OH are formed at the end of the reduction reaction with metal ions. At higher concentrations of **CuL0‐CuL3** (40 µM), a more efficient generation of ^•^OH radicals is expected, which explains the stronger fluorescence signal observed from TPA with Cu(II) complexes in the presence of a reducing agent in the TPA assay. However, these results illustrate that both the type of ROS and the site of radical generation play the essential role in the observed oxidative DNA cleavage activity. Therefore, it is of great importance to investigate the DNA binding affinity of the Cu(II) complexes in more detail.

### 2.3 DNA Interaction Studies

The binding affinity of nonfluorescent Cu(II) complexes **CuL1–CuL3** and their ligands as well as the nonalkylated Cu(II) oxacyclen toward calf thymus DNA (CT‐DNA) was investigated by the ethidium bromide (EtBr) displacement assay. CT‐DNA is a natural dsDNA that is commonly used in chemical, biochemical and medical research.^[^ ^49^ ^]^ EtBr is an aromatic planar cationic fluorophore, that intercalates between the base pairs of double‐stranded DNA with a binding constant (*K*_EtBr_) of 10^7^ M^−1^.^[^ ^50^ ^]^ The use of EtBr to probe drug‐DNA binding capability is based on the intercalation of EtBr a between the base pairs of double‐stranded DNA. The incubation of DNA with EtBr in solution leads to a DNA‐EtBr adduct with a strong fluorescence at 603 nm. After titration in this solution, competing DNA‐binding molecules, such as the here used Cu(II) complexes, can displace EtBr from the EtBr‐DNA adduct, resulting in the loss of the fluorescence.^[^ ^51^ ^]^ Changes in the fluorescence emission spectra during the titration of Cu(II) complexes **CuL1–CuL3** and the ligands alone with CT‐DNA (20 µM) and EtBr (1.3 µM) in MOPS (pH 7.4) were analyzed and visualized in Figure S5.2–S5.8, Supporting Information.

The emission spectra of the EtBr–DNA complex (black line) decrease almost proportionally with increasing concentration of the complexes **CuL1–CuL3**, whereby the alkylated complexes quench the fluorescence of the EtBr‐DNA compound at lower complex concentrations than the nonalkylated **CuL0**. Up to a complex concentration of ≈5 µM in the case of alkylated ligands and their Cu(II) complexes, a uniform quenching of the fluorescence emission of EtBr was observed. Additionally, a complex concentration in the range of ≈5–14 μM led to strong decrease in fluorescence intensity, indicating a stronger quenching effect by the complexes. This strong decrease might suggest the formation of complex‐DNA aggregates with increasing complex concentration, as also indicated by gel electrophoresis at micromolar concentrations (cf. 3.1). Sobell et al. report that the double helix of DNA must be flexible so that the intercalation of EtBr is possible.^[^ ^52^ ^]^ DNA condensation and aggregation results in the bending of DNA and thus to conformational changes within the double helix, leading to a loss of flexibility in the double‐strand structure of DNA. This favors the release of EtBr cations from the CT‐DNA and the binding equilibrium of EtBr is shifted in to the solution phase.^[^ ^53^ ^]^ It is assumed that DNA condensation decreases the affinity of the ethidium cation for DNA, whereby the displaced EtBr is no longer shielded from water or molecular oxygen by intercalation into the DNA bases, which may lead to additional quenching processes.^[^ ^54^ ^,^ ^55^ ^]^ Therefore, the release of EtBr at higher concentrations cannot be directly interpreted as an indication of the binding affinity. For this reason, complex concentrations <10 µM are taken into account to analyze the binding abilities of the compounds. Furthermore, studies have reported cases where EtBr was displaced from CT‐DNA by DNA‐condensation or compaction by cationic liposomes, resulting to the quenching of EtBr fluorescence intensity.^[^ ^54^ ^,^ ^55^ ^]^ This suggests that a similar mechanism may be responsible for the observed fluorescence quenching in the presence of the investigated Cu(II) complexes.

**Figure 6:**
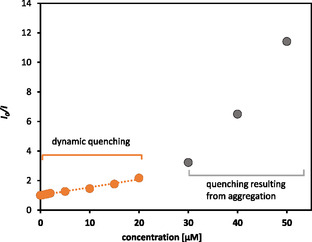
Stern–Vollmer plots of *I*_**0**_ */I* as a function of **CuL1** concentration, illustrating contributions from both static and aggregation‐induced quenching.

Quenching can occur through various molecular mechanisms, such as dynamic or static quenching, energy transfer and molecular rearrangement.^[^ ^56^ ^]^ Static quenching occurs when the fluorophore and quencher form a stable, nonfluorescent complex without any prior energy transfer between them. Consequently, the fluorescence lifetime remains unchanged. Dynamic quenching occurs when the excited fluorophore (EtBr‐DNA system) is deactivated by collisions with a quencher in solution without forming a stable complex. For the latter quenching process, a possible mechanism is photoinduced electron transfer (PET), in which the quencher acts as an electron acceptor or donor, leading to the deactivation of the excited state. This process is characteristic of redox‐active molecules or metal complexes, such as Cu(II).^[^ ^57^ ^,^ ^58^ ^]^ The differentiation between the two mechanisms which are reflected in Stern‐Volmer plots as shown in **Figure** **6**, where dynamic quenching results in a linear relationship and deviations from linearity indicate static or mixed quenching, can be achieved through the application of Equation (1), which is based on the bimolecular quenching rate constants (*K*_q_), where *τ*_0_ is the lifetime of the fluorophore (EtBr) without quenching.^[^ ^59^ ^,^ ^60^ ^]^

(1) $$K_{q}=K_{\mathrm{SV}}/\tau_{0}$$

If only one type of quenching is present, the extent of the decrease in fluorescence emission can be described by the classical Stern‐Volmer Equation (Equation 2).^[^ ^59^ ^,^ ^60^ ^]^

(2) $$\frac{I_{0}}{I}=1+K_{\mathrm{SV}}\left[Q\right]$$

where *I*_0_ represents the fluorescence emission in the absence and *I* in the presence of a competing compound, [*Q*] stands for the concentration of the competitor, and *K*_SV_ is the Stern‐Volmer constant.

As reference and state $\tau_{0}$ (EtBr) is 23 ns,^[^ ^61^ ^]^ so that the *K*_SV_ and *K*_q_ value for the investigated substances (**CuL0–CuL3**) can be calculated (**Table** **1**). The *K*_q_ values for all the complexes are in the same range of 10^12^ M^−1^s^−1^, which is close to the diffusion‐controlled limit, indicating dynamic quenching. The slightly increased values indicate the involvement of specific quenching mechanisms, such as PET or a metal‐induced energy transfer mechanism, in which PET occurs from the excited EtBr to the Cu(II) complex. This demonstrates that Cu(II) complexes not only influence the DNA structure but also interact with released EtBr via dynamic quenching.^[^ ^53^, ^54^, ^55^, ^56^, ^57^, ^58^, ^59^, ^60^, ^61^ ^–^ ^62^ ^]^ By plotting the ratio of the fluorescence intensities (*I*_0_ */I*) versus concentration [*Q*] the binding curve and the Stern–Volmer constant (*K*_SV_) can be determined.^[^ ^53^ ^]^ The binding constants (*K*_app_) of **CuL1–CuL3** with DNA were obtained using Equation (3). Here, [EtBr] is the concentration of EtBr and [*Q*]_50_ is the concentration of the investigated compound at which half of the intensity was achieved.^[^ ^59^ ^,^ ^60^ ^]^

(3) $$K_{\mathrm{EtBr}}\cdot \left[EtBr\right]=K_{\mathrm{app}}{\left[Q\right]}_{50}$$

Given that Cu(II) ions can quench the fluorescence of EtBr, we also examined the effect of Cu(NO_3_)_2_ in solution on their quenching ability (Figure S5.1, Supporting Information). Titration of Cu(II) in the absence of a ligand results in only a weak enhancement of the quenching effect. Therefore, the presence of the ligand is crucial for strong quenching.

**Table 1:** Evaluated binding parameters obtained for the interactions of the Cu(II) complexes and their ligands with CT‐DNA (calculated Stern–Volmer constants *K*_SV_ and corresponding binding constants *K*_app_ toward CT‐DNA).

Compound	*K*_SV_ [M^−1^]	*K*_app_ [M^−1^]	*K*_q_ [M^−1^ s^−1^]
EtBr		10^7^	
Cu(NO_3_)_2_	1.7 × 10^4^	2.2 × 10^5^	8.4 × 10^11^
CuL0	5.4 × 10^4^	6.9 × 10^5^	2.7 × 10^12^
CuL1	5.4 × 10^4^	7.1 × 10^5^	2.7 × 10^12^
CuL2	5.7 × 10^4^	7.5 × 10^5^	2.9 × 10^12^
CuL3	8.9 × 10^4^	1.2 × 10^6^	4.4 × 10^12^
L1	3.6 × 10^4^	4.7 × 10^5^	1.8 × 10^12^
L2	3.6 × 10^4^	4.6 × 10^5^	1.8 × 10^12^
L3	3.1 × 10^4^	3.9 × 10^5^	1.5 × 10^12^

The values for the free ligands and the complexes show, that they both compete for DNA binding sites and so displace EtBr from the DNA. However, the *K*_app_ values for the ligands are lower than for Cu(II) complexes, and a correlation between binding constants and chain length of the alkyl substituents is observed. The data suggests that the interaction of Cu(II) complexes with CT‐DNA is stronger than that of the respective ligands.

*K*_app_ values of **CuL1–CuL3** are in the same order of magnitude (in the range of 10^5^), which indicates a similar binding affinity to DNA. It is noticeable that the binding constant of **CuL3** is about 2‐fold greater than that of **CuL1**. The increased *K*_app_ values can be attributed to the presence of the alkyl chain, which interacts with the DNA grooves (presumably major groove, vide supra) and effectively displaces EtBr. DNA intercalation typically occurs with planar aromatic systems due to favorable π–π interactions with the aromatic nucleobases, as in the case of EtBr^[^ ^63^ ^]^ or as observed in the case of AQ‐substituted Cu(II)‐cyclen complexes, where the corresponding *K*_app_ values were in the range of 10^7^ M^−1^.^[^ ^35^ ^]^ Since the investigated complexes **CuL1–CuL3** lack aromatic substituents, classical intercalation can be excluded, and as suggested by the MG gel electrophoresis experiment, interaction with DNA is rather likely a groove binding. As an additional mode of interaction electrostatics surely play a role:^[^ ^64^ ^]^ the cationic core Cu(II) of the complexes exerts an electrostatic attraction on the anionic phosphate backbone of DNA.^[^ ^63^ ^]^

The interaction between small molecules and DNA can lead to morphological DNA changes, which can be observed using circular dichroism (CD) spectroscopy. The conformational changes of the DNA backbone depend on the type of binding and on the extent of the compound–DNA interaction.^[^ ^65^ ^,^ ^66^ ^]^ For instance, the intercalation of small molecules into the DNA double strand leads to the stabilization of a right‐handed B‐conformation of the CT‐DNA and results in changes in the CD spectrum, in particularly an increase in the positive band and a decrease in the negative band at, indicating enhanced base stacking interactions. At the same time the groove binding and the electrostatic interaction has no influence on the base stacking and therefore do not induce comparable changes in the CD spectrum.^[^ ^65^, ^66^ ^–^ ^67^ ^]^

The experiment was conducted with CT‐DNA, which is present in aqueous solution in the B‐DNA form.^[^ ^67^ ^]^ The results of the CD spectroscopic analysis of the Cu(II) complexes are represented in Figure S6.1, Supporting Information. The characteristic spectrum of B‐DNA form was observed (black line) for CT‐DNA, with a positive band at 275 nm due to stacking of the nucleobases and a negative band at 245 nm, which occurs due to the right–handed helicity of the DNA double helix.^[^ ^65^ ^,^ ^66^ ^]^ When titrating **CuL1–CuL3** into the CT‐DNA solution, the recorded CD spectra show a very similar profile to the CT‐DNA reference. The intensity of the positive band (≈275 nm) slightly decreases with increasing **CuL1–CuL3** concentration, while the negative band (≈245 nm) increases in intensity with the addition of the alkylated complexes, suggesting an enhanced binding affinity by extending the chain length of the alkyl substituents. The nonalkylated Cu(II) complex **CuL0** does not interact with DNA, as both bands in the spectrum remain unchanged (Figure S6.1.1, Supporting Information). CD spectroscopic analysis showed that the **CuL1–CuL3 complexes** generate relatively weak distortions of both characteristic CT‐DNA bands. Thus, the complexes must bind to the DNA via a nonintercalative binding mode, which accounts for electrostatic interactions and/or groove binding mode with the DNA helix, that stabilizes the right‐handed B form of DNA.^[^ ^68^ ^]^ Possibly, groove binding is favored for these complexes due to hydrophobic interactions between the alkyl chains and the hydrophobic DNA strand interior. Extraction of the CD values at 245 and 275 nm as a function of complex concentration (Figure S6.1.2, Supporting Information) shows that at higher complex concentrations (≥20 µM), the intensity of both positive and negative CD signals by **CuL1–CuL3** decrease rapidly upon increment of concentration. The progressive loss of intensity of CD bands may be caused by conformational changes of DNA upon binding with the **CuL1–CuL3** complexes. This could result from DNA condensation/aggregation, leading to secondary structural changes, or it may indicate precipitation.^[^ ^69^ ^]^ The latter was, however, not visible by naked eye during the experiment. Based on the results from CD spectroscopy, gel electrophoresis and EtBr fluorescence spectroscopy, we can conclude that the amphiphilic Cu(II) oxacyclen complexes primarily bind to DNA in a groove‐binding mode, with DNA complex aggregation occurring at higher complex concentrations.

The DNA‐binding nature of the complexes **CuL0–CuL3** was also determined by thermal denaturation, as the interaction of the molecules with double‐stranded DNA can influence the melting temperature (*T*_m_).^[^ ^70^ ^,^ ^71^ ^]^ At the melting temperature, half of the double helix denatures into the single‐stranded DNA. Intercalation of molecules between the DNA base pairs leads to stabilization of the double helix, which results in a strong increase in *T*_m_, as much as 5–8 °C, while the nonintercalative binding causes no obvious variation of *T*_m_.^[^ ^72^ ^]^ The melting temperature *T*_m_ of the DNA was monitored by UV/VIS spectroscopy by observing the change in absorbance at 260 nm as a function of temperature. Figure S7, Supporting Information, shows the normalized melting profile of CT‐DNA alone and in the presence of copper complexes, and the complex‐induced changes of the melting temperatures (Δ*T*_m_) are listed in Table S7, Supporting Information. The melting temperature *T*_m_ of CT‐DNA is hardly increased in the presence of **CuL2** and **CuL3**. However, the Cu(II) complex with the shortest alkyl chain **CuL1** leads to a significant increase in temperature with Δ*T*_m_ 1.13 °C as well as for **CuL0**. This results in stabilization of the DNA in the order **CuL1** > **CuL3** ≥ **CuL2.** A very small change in *T*_m_ supports the groove binding nature and electrostatic interactions^[^ ^70^ ^,^ ^71^ ^]^ of **CuL1–CuL3**. This is consistent with the previously conducted experiments.

### 2.4 Analysis of DNA Aggregates

To obtain further information on the effect of the complexes on the morphology of the complex‐DNA aggregates atomic force microscopy (AFM) was used. The advantage of this method is the ability to visualize 3D structures of the objects being examined.^[^ ^73^, ^74^ ^–^ ^75^ ^]^ For this purpose, linearized pBR322 plasmid DNA was incubated with the Cu(II) complexes, loaded onto a silica chip and subsequently observed by AFM. In Figure S8.1.–S8.4., Supporting Information, representative AFM images of these experiments are presented, illustrating the morphological changes of linearized pBR322 DNA under different conditions.

Figure S8.1., Supporting Information, shows that the DNA appears in a linearized form when observed alone, which is characteristic of uncondensed DNA. It is distributed over the entire surface. No morphological changes were observed with **CuL1** (30 µM). The only molecules present were the free DNA molecules. After addition of 3 µM of **CuL2**, the DNA molecules were induced to form nearly globular particles with a diameter of ≈200 nm (Figure S8.3., Supporting Information left). As shown in Figure S8.3., Supporting Information right, by increasing the concentration of the complex to 30 µM, all DNA molecules condensed into larger particles. A similar condensation phenomenon was observed when the DNA was incubated with the **CuL3** complex. In this case, the DNA appeared in a more condensed form, which can be seen as a dense aggregate from which the DNA loops and additionally free DNA molecules emerge (at the complex concentration of 3 µM in Figure S8.4., Supporting Information left).^[^ ^76^ ^,^ ^77^ ^]^ When the concentration of the complex was increased to 30 µM, fewer aggregates became traceable on the surface. It is conceivable that higher concentrations lead to the formation of bulkier DNA aggregates, which are less distributed over the surface and it becomes less likely to image them by AFM. Also with this method, it could be confirmed that the DNA condensation induced by Cu(II) complexes is concentration‐dependent, whereby the length of the alkyl substituents has an influence on the formation and size of the aggregates.

Dynamic light scattering (DLS) is widely used to determine the hydrodynamic diameter of macromolecules in a liquid environment.^[^ ^78^ ^]^ We applied this method to measure the particle size of aggregates formed by pBR322 plasmid DNA and the Cu(II) complexes in MOPS buffer by maintaining the same reaction conditions used for the gel electrophoresis studies (cf. 3.1). We chose a concentration of 30 µM of the complex for the experiment, as DNA condensation was observed at this concentration in the presence of **CuL2** and **CuL3**, as determined by AFM. The experimental results are presented in Figure S11.1–S11.3., Supporting Information, as intensity‐based hydrodynamic size‐distributions. In the presence of **CuL1**, a dominant peak with a hydrodynamic diameter of 615 nm was detected, indicating that aggregation could start immediately after an incubation time of 2 min. At longer reaction times, inconsistent results are observed (data not shown), suggesting the possible formation of asymmetric aggregates that cannot be detected by DLS.^[^ ^78^ ^]^ In the case of complex **CuL2**, the hydrodynamic diameter was 295 nm, while a value of 142 nm was determined for **CuL3** (Figure S9.1.–9.3., Supporting Information). Increasing alkyl chains might cause stronger hydrophobic interactions leading to a tighter packing of the aggregates in solution.^[^ ^79^ ^,^ ^80^ ^]^ It should be noted that the bandwidth increases with increasing length of the alkyl substituents, reflecting the size distribution of the aggregates. It is possible that complexes with longer alkyl chains promote the formation of smaller aggregates. The aggregates formed by **CuL1** under the investigated conditions have a relatively more homogeneous size, which could indicate higher stability. Additionally, a peak at 5560 nm is visible for complexes **CuL1** and **CuL3** with low intensity, which probably can be attributed to dust or similar. To summarize, aggregate formation could be confirmed using both AFM and DLS methods. AFM showed increasing aggregate size with longer alkyl chains, while DLS indicated a decrease in hydrodynamic diameter, which includes both the particles and the surrounding hydration shell. It should be emphasized that the hydration shell plays a central role in the structure and dynamics of biomolecules, while the size and shape of the aggregates can significantly influence their water binding capacity.^[^ ^81^ ^]^ Additionally, it should be noted that different incubation times were used, which could also have influenced the results. Amphiphilic Cu(II) complexes induce DNA condensation, appearing larger in AFM due to surface adsorption but more compact in DLS due to tighter packing in solution. This discrepancy can be attributed to the different weighting factors of the two methods, especially for samples with a heterogeneous particle size distribution.^[^ ^79^ ^,^ ^80^ ^,^ ^82^ ^]^ While DLS provides intensity‐weighted distributions in solution, thereby disproportionately emphasizing larger particles, AFM enables a direct imaging‐based analysis of particle sizes on a solid substrate.

### 2.5 Molecular Dynamics Simulations

**Figure 7:**
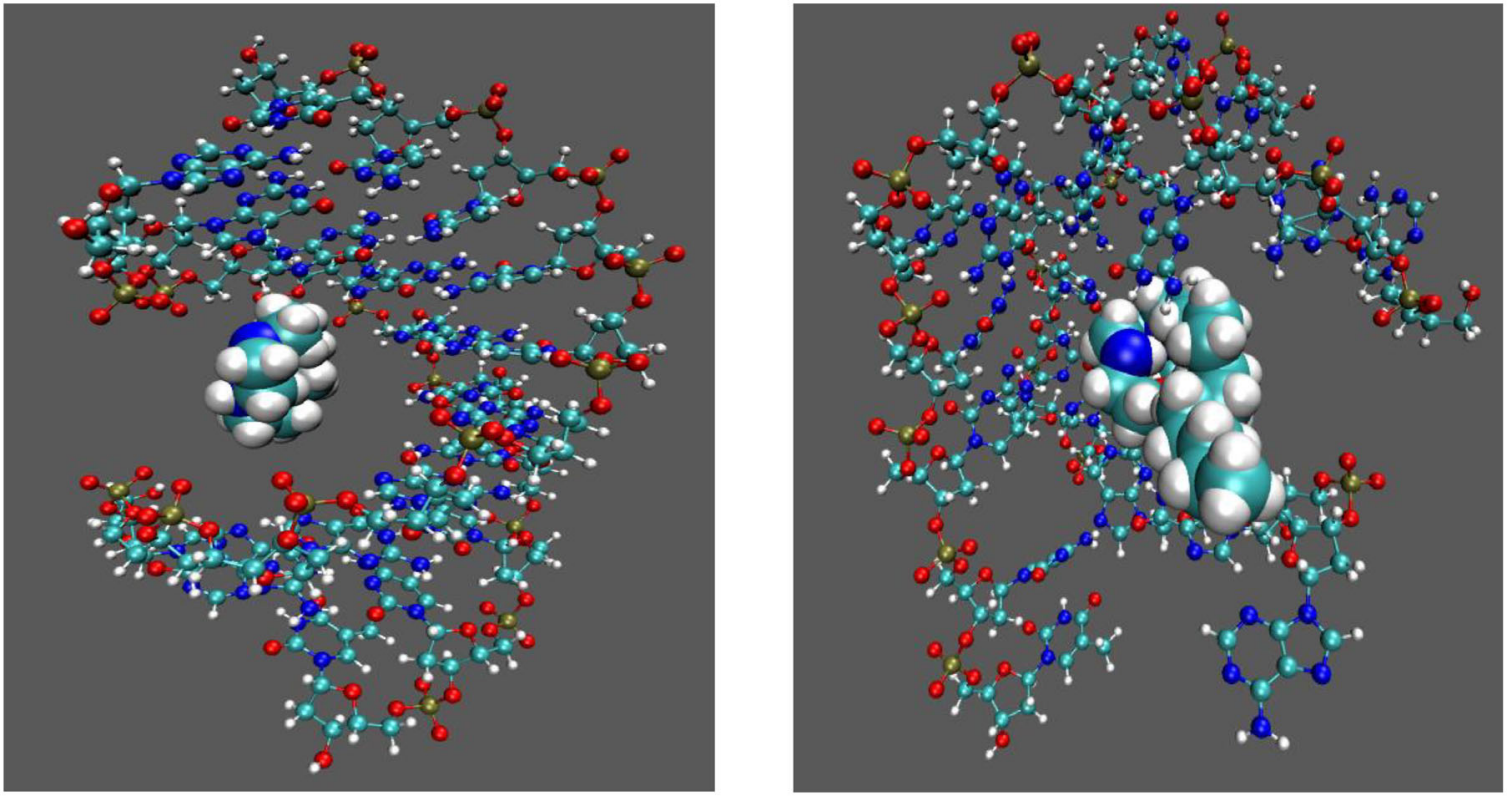
DNA/**L0** (**left**) versus DNA/**L1** (**right**) with the alkyl chain being exposed to the solvent water.

Molecular dynamics simulations showed that the investigated ligands **L0–L3** form thermodynamically stable complexes with DNA, with DNA‐ligand binding energies as obtained as the sum of electrostatic and LJ pairwise interactions in the negative range, indicative of strong affinity. While the simulations were performed with the free ligands (without Cu(II) ions, which were present in the experimental complexes), due to demanding calculation times the simulations including the copper metal ion are challenging, because the force‐field for the interaction between metal ions and DNA is not readily available and thus has to be developed. Moreover, the metal ion is present in all four cases and we want to only explore the effect of alkyl chain length on the DNA ligand binding/DNA aggregation. For this reason, we performed the simulations without the central copper ion. We assume that the binding mode is consistent, and inclusion of the metal center would likely just further increase the binding strength due to additional electrostatic contributions. A key difference between alkylated (here, e.g. **L1**) and the nonalkylated ligand (**L0**) is that the alkyl chains remain exposed to the solvent, creating a hydrophobic surface prone to interactions with alkyl groups from ligands bound to neighboring DNA duplexes (**Figure 7**). This provides a plausible molecular basis for the aggregation observed experimentally by gel electrophoresis and AFM. Moreover, simulations indicated a destabilization of the DNA duplex in the presence of alkylated ligands (e.g., **L1**, Figure 7 right), including local strand separation, which would expose single‐stranded regions and further promote nonspecific intermolecular aggregation. Together, these findings support two complementary pathways toward DNA condensation: (i) hydrophobic clustering of solvent‐exposed alkyl chains, and (ii) destabilization of the duplex leading to singles‐stranded ‘sticky’ segments. Both mechanisms align well with the experimental evidence and offer a coherent explanation for the DNA aggregation phenomena observed.

## 3 Conclusions

Cu(II) complexes with monoalkylated oxacyclen ligands (1‐oxa‐4,7,10‐triazacyclododecane) were investigated regarding their nuclease activity toward plasmid DNA, whereby the complexes differed in the length of the alkyl substituents. It could be shown, that **CuL1–CuL3** show a DNA cleavage activity at complex concentration lower than 10 µM. Furthermore, it was observed that with increasing concentration of the complex and increasing length of the alkyl chain, DNA‐complex adducts are formed. ROS quenching experiments and BNPP cleavage studies showed that the mechanism of the DNA cleavage for **CuL1–CuL3** is both hydrolytic and oxidative. As for the ROS, fluorescence assays show that ^•^OH and H_2_O_2_ are involved in the oxidative mechanism.

The influence of the alkyl chain length on the binding of the complexes with CT‐DNA was thoroughly examined using various spectroscopic techniques. UV/VIS (DNA melting curves), CD and fluorescence (EtBr displacement assay) spectroscopy indicated that the complexes bind to DNA through both electrostatic interactions and groove binding, which was further confirmed in the groove binding experiment with methyl green. In general, a longer alkyl chain results in a stronger binding affinity. The aggregation behavior of pBR322 plasmid DNA in the presence of amphiphilic Cu(II) complexes was investigated using both AFM and DLS. The results from both techniques consistently indicate the formation of aggregates upon complex binding, with DNA aggregation occurring at lower concentrations as the alkyl chain length increased. The molecular dynamics simulations also show a propensity of DNA aggregation driven by duplex destabilization and hydrophobic clustering in presence of long chain alkyl groups in the ligand.

## 4 Experimental Section

4.1

4.1.1

##### Materials and Instrumentation

The complexes **CuL0** (Cu(II)‐1‐oxa‐4,7,10‐triazacyclododecane) (used for comparison only in the BNPP assay, terepthalate (TPA)/ pentafluorobenzenesulfonyl fluorescein (PBSF) and EtBr fluorescence experiments), **CuL1** (Cu(II)‐7‐dodecyl‐1‐oxa‐4,7,10‐triazacyclododecane) and **CuL2** (Cu(II)‐7‐hexadecyl‐1‐oxa‐4,7,10‐triazacyclododecane) were prepared according to methods published in the literature.^[^^29,30]^ **L3** (7‐octadecyl‐1‐oxa‐4,7,10‐triazacyclododecane) and its corresponding Cu(II) complex **CuL3**, both of which have not been previously synthesized or characterized according to the literature, were obtained using an analogous procedure (cf. S‐0). The Cu(II) complex stock solutions were prepared in accordance with the prior instructions.^[^^30]^

Chemicals and solvents were purchased from Alfa Aesar, Carl Roth, Cayman Chemicals, Fisher Scientific, Fluka, Lonza, Sigma–Aldrich and were used without further purification. UV/VIS measurements were carried on an Agilent Cary 100 Bio UV/VIS spectrophotometer. Fluorescence emission spectra were recorded on an Agilent Cary Eclipse fluorescence spectrophotometer.

##### DNA Cleavage Activity Studies

The nuclease activity of the Cu(II) complexes toward pBR322 plasmid DNA was investigated by gel electrophoresis. All experiments were performed in triplicate to provide reproducibility and to determine a standard deviation.

A mixture of Cu(II) complexes **CuL1**, **CuL2** or **CuL3** (5–50 µM or in varying concentrations), respectively, and pBR322 plasmid DNA (0.025 µg/µL) in (*N*‐morpholino)propanesulfonic acid (MOPS) buffer (50 mM, pH 7.4) in the absence or presence of L‐ascorbic acid as reducing agent (0.32 mM) was incubated for 2 h (or 24 h) at 37 °C and 500 rpm. The reference sample was prepared using pBR322 plasmid DNA and ascorbate in MOPS buffer. The total reaction volume amount was 8 µL. After incubation, 1.5 µL of loading buffer (1.2 mM saccharose and 3.7 mM bromophenol blue in deionized water) was added to each sample and afterwards loaded into the pockets of the agarose gel (1% agarose in (0.5x) Tris‐boric acid‐EDTA (TBE) buffer and EtBr (0.2 µg mL^−1^)). Electrophoresis was carried out with an electrophoresis unit (Carl Roth; power supply: consort EV243) for 2 h at 40 V. DNA bands were visualized by fluorescence imaging of EtBr with Bio‐Rad GelDoc EZ Imager. Data analysis was performed using Bio‐Rad's Image Lab software (version 3.0). The resulting three main bands were identified as the supercoiled (form I), the open circular (form II) and the linear (form III) DNA. The intensity of the bands was compared to a reference DNA. A correction factor of 1.22 was applied in case of the supercoiled DNA (Form I) due to the affinity of EtBr to the supercoiled plasmid DNA.^[^ ^83^ ^]^ The intensity of the bands was based on the reference DNA.

##### ROS Quenching Assay

For the detection of ROS the Cu(II) complex (20 µM), pBR322 plasmid DNA (0.025 µg µL^−1^), L‐ascorbic acid (0.32 mM) in MOPS buffer (50 mM, pH 7.4) and one of the following ROS scavengers: DMSO (200 mM), NaN_3_ (10 mM), pyruvate (2.5 mM), and superoxide dismutase (SOD) (625 U mL^−1^) were incubated for 2.5 h at 37 °C and 500 rpm. Gel electrophoresis was carried out according to the procedure described above (2.2).

##### Detection of Hydroxyl Radicals by Fluorescence Using TPA

The fluorescence experiments were carried out at room temperature in MOPS buffer (50 mM, pH 7.4). The samples were prepared from MOPS buffer (50 mM, pH 7.4), TPA (0.5 mM), L‐ascorbic acid (0.25 mM), DMSO (200 mM), and the respective Cu(II) complex (40 µM). This mixture was incubated for 2.5 h at 37 °C, 500 rpm and fluorescence spectra were then recorded in a range of 350–550 nm. The excitation wavelength *λ*_ex_ was 320 nm (slit width 5 nm) and the measuring speed was 100 nm min^−1^. The photomultiplier voltage was adjusted to 850 V.

##### Detection of Hydrogen Peroxide by Fluorescence Using PBSF

The samples included PBSF (25 µM), L‐ascorbic acid (0.25 µM), pyruvate (2.5 mM), the respective Cu(II) complex (40 µM) in MOPS buffer (50 mM, pH 7.4) with a total volume of 500 µL. The samples were incubated for 2.5 h at 37 °C and 500 rpm. The emission spectra were recorded in a range of 490–600 nm with an excitation wavelength *λ*_ex_ of 485 nm (slit width 5 nm) and a scan rate of 100 nm min^−1^. The photomultiplier voltage was adjusted to 560 V.

##### Ethidium Bromide Displacement Assay

The Cu(II) complexes and their ligands were titrated (0.5–50 µM) into a solution of calf thymus DNA (CT‐DNA) (20 µM), MOPS buffer (50 mM, pH 7.4), and EtBr (1.3 µM) in deionized water, with a total volume of 1000 µL. The fluorescence spectra were recorded in the range 540–730 nm. The excitation wavelength (*λ*_ex_) was set to 518 nm (slit width 5 nm) with a scan rate of 100 nm min^−1^, the photomultiplier voltage was adjusted to 950 V.

##### Methyl Green Displacement Assay

The fluorescence spectra with the fluorescent probe methyl green (1 µM) were performed by keeping the DNA concentration constant (50 µM) in 50 mM MOPS buffer (pH 7.4) while varying the **CuL1** concentration (0–20 µM). The experimental parameters were as follows: *λ*_ex_ = 633 nm (slit width 10 nm); scan rate of 100 nm min^−1^; emission in the range of 650–740 nm. The photomultiplier voltage was adjusted to 950 V.

##### DNA Melting Point Determination

The DNA melting temperature was determined by the means of UV/VIS spectroscopy. The change in absorbance of an aqueous solution of CT‐DNA (50 µM), Cu(II) complex (2.5 µM) in MOPS buffer (50 mM, pH 7.4) was determined at 260 nm. The melting curves were recorded in 1 °C steps in the range of 60–95 °C with a heating rate of 0.5 °C min^−1^. For a better visualization, the individual absorbances were normalized and illustrated as a function of the temperature.

##### Bis(4‐Nitrophenyl) Phosphate Assay

A mixture of BNPP (0.08 mM) in MOPS buffer (50 mM, pH 7.4) was incubated with the respective Cu(II) complex (0.4 mM) for 2 and 24 h at 37 °C and 500 rpm. BNPP (0.08 mM) in MOPS buffer (50 mM, pH 7.4) was used as a negative control. The volume of each sample was adjusted using deionized water to 500 µL. A background measurement was performed before each individual measurement was conducted (complex without BNPP). Following the incubation, the samples were analyzed in the range of 200–500 nm.

##### CD Spectroscopy

CD spectra were recorded under a constant stream of nitrogen at 25 °C in Hellma fluorescence cuvettes (10 mm). To the aqueous solution consisting of CT‐DNA (100 mM) in MOPS buffer (50 mM, pH 7.4) the Cu(II) complexes were titrated in 5 µM steps (5–15 µM). The volume of the samples was adjusted to 1000 µL. The CD spectra were measured in the range 220–320 nm with a measuring speed of 100 nm min^−1^ collecting data points every 0.1 nm. The background was measured with the complexes alone without CT‐DNA.

##### Imaging by AFM

pBR322 plasmid DNA was linearized by digestion with NDE I restriction endonuclease. ***Linear (form III)*** pBR322 plasmid DNA was purified with the of GenElute GelExtraction Kit (Sigma Aldrich) from the agarose gel. The Cu(II) complexes **CuL1–CuL3** (3–30 µM) were dissolved in MilliQ water and mixed with DNA (20 ng), KCl (5 mM) and MgCl_2_ (5 mM) in HEPES buffer (pH 7.4) and incubated for 5 min at room temperature. The incubation solution was loaded directly onto freshly cleaned silicon chips. After 2 min incubation, the silicon chips were gently rinsed with 1 mL of MilliQ water and immediately blown dry with compressed air. The samples were imaged using an atomic force microscope (HORIBA OmegaScope) operating in tapping mode (AC mode) in air with a scan rate of 1 Hz. TAP 150 AI‐G tips were used, which have a resonance frequency of 150 kHz and a force constant of 5 N m^−1^.

##### DLS

DLS measurements were carried out using a Dawn Heleos light scattering spectrometer (Wyatt Corporation) with a maximum wavelength *λ* = 320 nm to investigate the condensation of DNA (pBR322 plasmid DNA, 0.025 µg µL^−1^) in the presence of 30 µM of Cu(II) complexes in a 50 mM MOPS buffer solution (pH 7.4) at 37 °C. Reaction conditions were the same as for the gel electrophoresis assay for the sake of comparison. Data were collected at a scattering angle of 90°.

##### Molecular Dynamics Simulation

All simulations of DNA and **L0–L3** complex without the central Copper metal ion were performed using Gromacs 2022.4 version.^[^ ^84^ ^]^ The initial configuration of DNA was taken from protein data bank (PDB: 440D). The DNA and **L0–L3** complex interactions were modeled using AmberGS forcefield^[^ ^85^ ^]^ and solvent water molecules using SPC water model as implemented in Gromacs. The van der Waals interactions were computed within a cutoff distance of 10 Å and electrostatics were accounted for using PME (particle mesh Ewald) algorithm. The DNA and **L0–L3** complex systems were equilibrated in NVT and NPT ensemble for a duration of 1000 ps at 300 K and 1 atm pressure using a timestep of 1 fs. The temperature and pressure were kept constant using velocity rescaling thermostat and Berendsen barostat. Finally, the DNA‐complex interactions were analyzed based on the trajectory obtained from NVE ensemble simulations of 1000 ps. To explore the DNA aggregation/condensation, the bond constraints on DNA and complex were relaxed in NVE simulations.

## Conflict of Interest

The authors declare no conflict of interest.

## Supporting information

Supplementary Material

## Data Availability

The data that support the findings of this study are available in the supplementary material of this article.
