# Supplementary material for: Amphiphilic Cu(II) Oxacyclen Complexes: From Oxidative Cleavage to Condensation of DNA
Source: Chembiochem. 2026 Jan 22;27(2):e202500477. doi: 10.1002/cbic.202500477 (PMC12828249; doi:10.1002/cbic.202500477)
Supplement: Supplementary file 1 — Supplementary Material [file CBIC-27-e202500477-s001.pdf]

**Electronic Supplementary Information**

Accompanying the manuscript

# Amphiphilic Cu(II) Oxacyclen Complexes: From Oxidative Cleavage to Condensation of DNA

**Olga Verbitsky,[a] Sebastián Hinojosa,[b] Amr Mostafa,[a] Deepak Ojha,[a] Ilko Bald,[a] Nora Kulak[a,*]**
a Institute of Chemistry, University of Potsdam, Karl-Liebknecht-Str. 24/25, 14476, Potsdam, Germany
b Institute of Chemistry and Biochemistry, Freie Universität Berlin, Fabeckstr. 34/36, 14195 Berlin, Germany

## List of contents

**S-0. Synthesis and characterization of complex CuL3**
**S-1. Nuclease activity in the presence of reducing agent**
**S-2. Nuclease activity in the absence of reducing agent**
**S-3. Bis(4-nitrophenyl) phosphate assay**
**S-4. Detection of hydroxyl radicals and hydrogen peroxide**
**S-5. Ethidium bromide displacement assay**
**S-6. Circular dichroism spectroscopy**
**S-7. DNA melting point determination**
**S-8. Atomic force microscopy**
**S-9. Dynamic light scattering**

## S-0. Synthesis and characterization of complex CuL3

### Synthesis of 7-octadecyl-1-oxa-4,7,10-triazacyclododecane (L3)

7-octadecyl-4,10-ditosyl-1-oxa-4,7,10-triazacyclododecane (1.41 g, 1.928 mmol) was dissolved with phenol (3.63 g, 38.57 mmol) in 69 mL 33% HBr in acetic acid solution. The red mixture was stirred for 2 d at 90 °C. Afterwards the mixture was brought to room temperature. To the mixture 190 mL diethyl ether were added. The precipitate formed was filtered and washed with 95 mL diethyl ether. The precipitate was then portioned in 95 mL 2.5 N NaOH and 95 mL dichloromethane. After separation, the aqueous phase was further extracted three times with 30 mL dichloromethane. Subsequently, the organic layers were combined and dried over $Na_2SO_4$. After concentration under reduced pressure a white solid was obtained (0.36 g, 45%). HRMS (ESI) calcd. for $[M+H]^+$ 426.4418; found 426.4470; $^1$H NMR ($CDCl_3$, 400 MHz): $\delta$=3.61 (t, *J*=4.8 Hz, -C*H*$_2$OC*H*$_2$-(macrocycle), 4H), 2.77 (t, *J*=4.8 Hz, -C*H*$_2$CH$_2$OCH$_2$C*H*$_2$-(macrocycle), 4H), 2.62 (dd, *J*=6.5, 3.2 Hz, -NCH$_2$C*H*$_2$NH-(macrocycle), 4H), 2.54 (dd, *J*=6.5, 3.2 Hz, -NC*H*$_2$CH$_2$NH-(macrocycle), 4H), 2.39 (t, *J*=7.3 Hz, -CH$_2$C*H*$_2$N-, 2H), 1.42 (m, -C*H*$_2$CH$_2$N, 2H), 1.24 (m, alkyl chain, -N*H*, 30H), 0.86 (m, -C*H*$_3$, 3H) ppm. $^{13}$C NMR ($CDCl_3$, 126 MHz): δ=66.58 (-*C*H$_2$O*C*H$_2$-(macrocycle)), 55.25 (-CH$_2$*C*H$_2$N-(chain)), 52.27 (-NC*H*$_2$CH$_2$NH (macrocycle)), 46.66(-*C*H$_2$CH$_2$OCH$_2$*C*H$_2$- (macrocycle)), 44.91 (-NCH$_2$*C*H$_2$NH (macrocycle)), 32.05 (CH$_3$CH$_2$*C*H$_2$-(chain)), 29.83, 29.82, 29.80, 29.78, 29.76, 29.68, 29.49 (chain) 27.60, 27.52 (-*C*H$_2$CH$_2$CH$_2$N- (chain)), 22.82 (CH$_3$*C*H$_2$- (chain)), 14.25 (-*C*H$_3$) ppm. $C_{26}H_{55}N_3O$: calcd. C 73.35, H 13.02, N 9.87; found C 73.29, H 12.96, N 9.94.

### Synthesis of Cu(II)-7-octadecyl-1-oxa-4,7,10-triazacyclododecane (CuL3)

A solution of $Cu(NO_3)_2$ trihydrate (88 mg, 0.364 mmol) in 5 mL MeOH was added to a solution of the ligand (100 mg, 0.239 mmol) in 5 mL MeOH. During the addition of the metal salt the solution containing the ligand turned deep blue. After cooling to room temperature, the solution was stored at -20 °C. The next day the blue precipitate formed was filtered and dried *in vacuo* (66.7 mg 46%). HRMS (ESI) calcd. for $[M+NO_3^-]^+$ 550.3514; found 550.3552. $[M+Cl^-]^+$ 523.3324; found 523.3363. $C_{26}H_{55}CuN_5O_7$: calcd. C 50.92, H 9.04, N 11.42; found C 50.99, H 9.09, N 11.47.

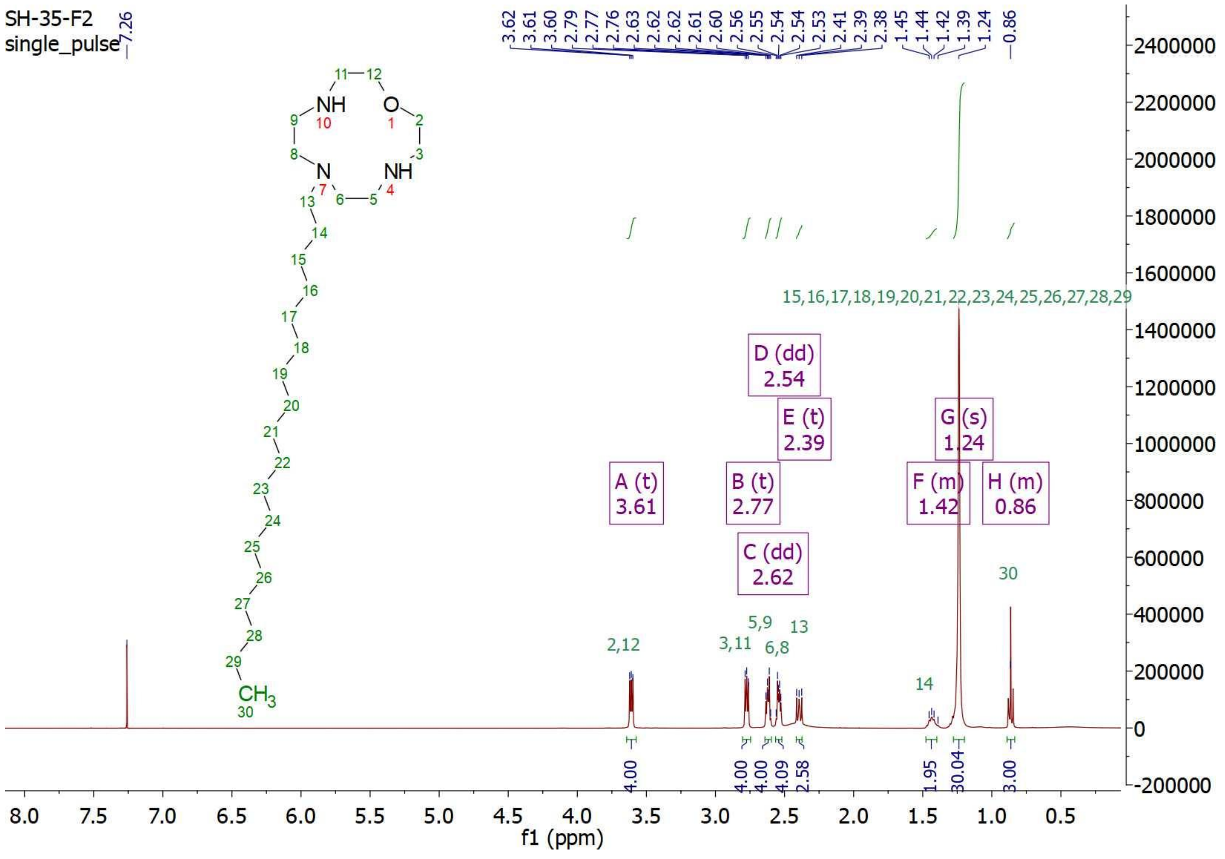


**Figure S0.1.** $^1$H NMR spectrum of **L3** in $CDCl_3$.

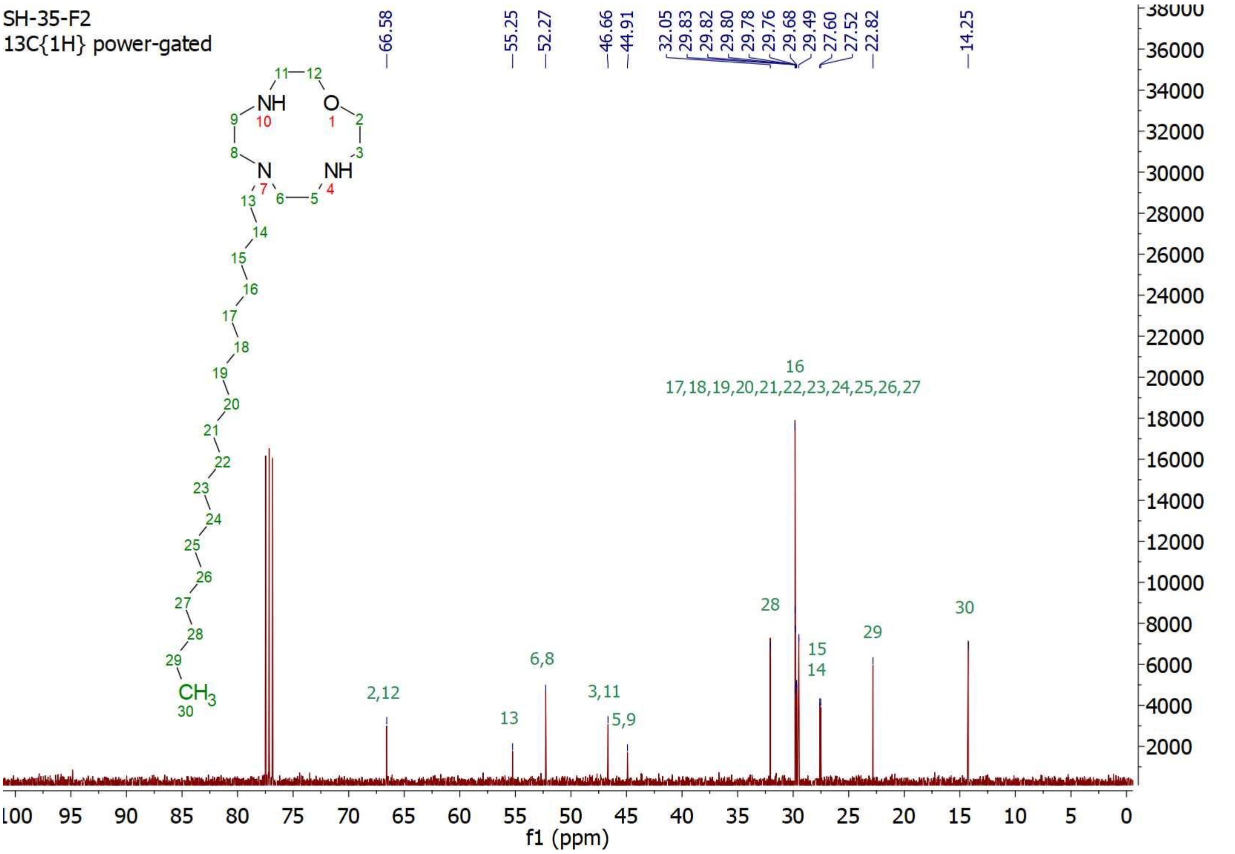


**Figure S0.2.** $^{13}C$ NMR spectrum of **L3** in $CDCl_3$.

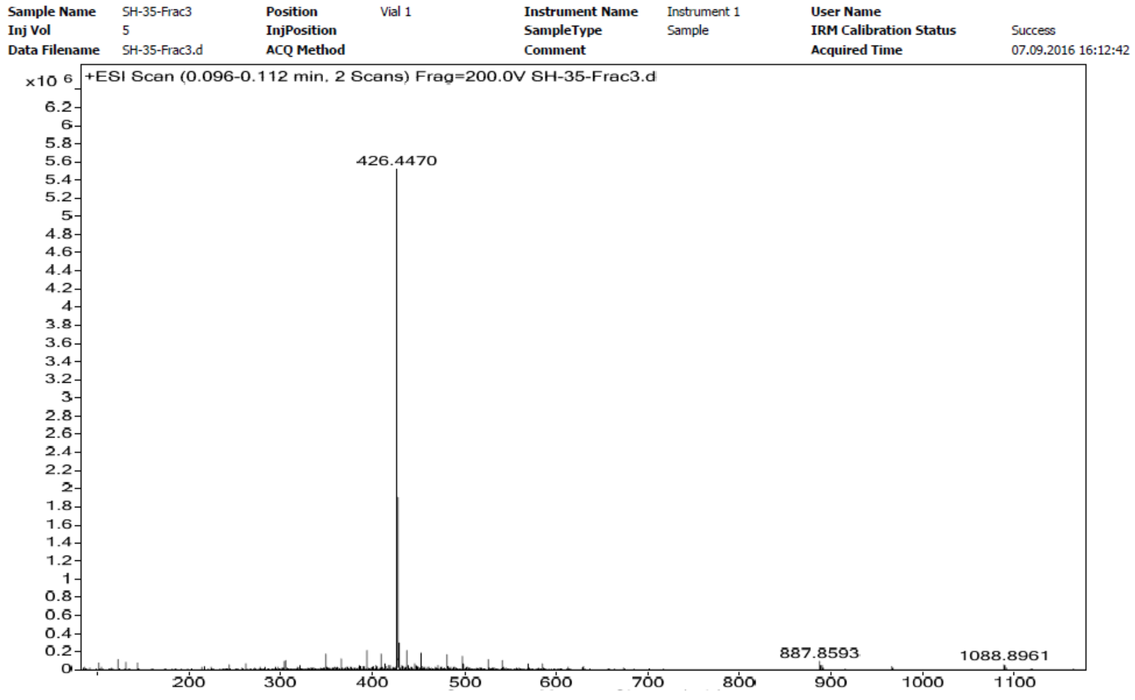


**Figure S0.3.** ESI mass spectrum of **L3** in methanol.

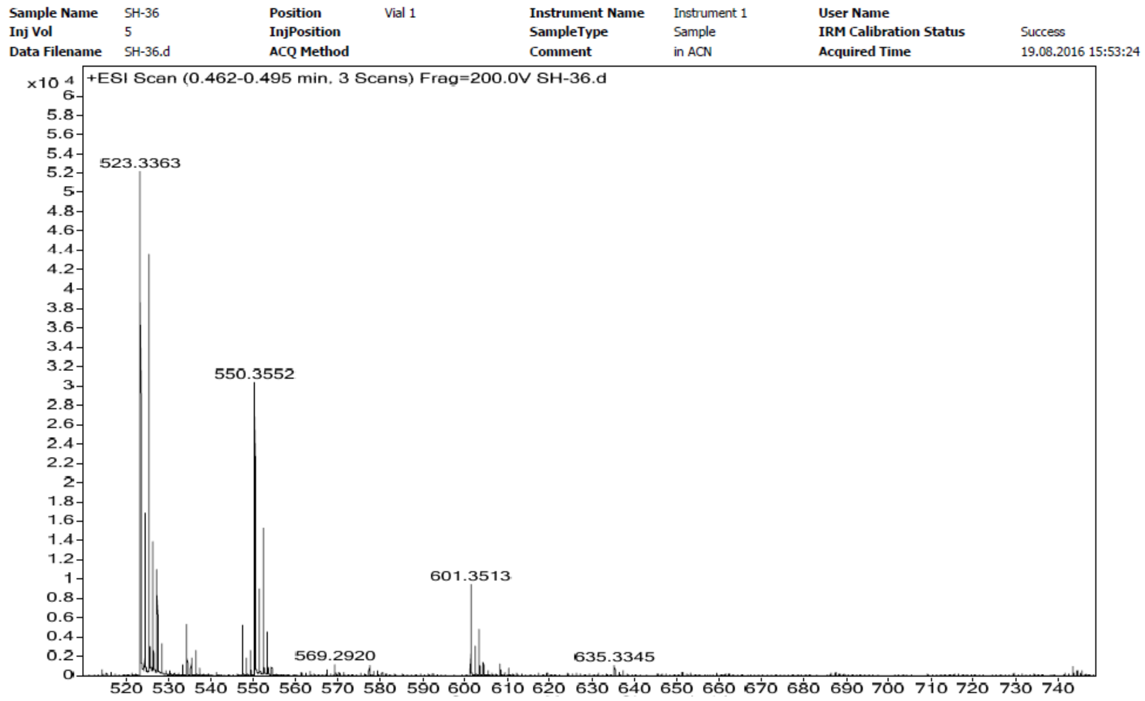


**Figure S0.4.** ESI mass spectrum of **CuL3** in acetonitrile.

## S-1. Nuclease activity of the complexes

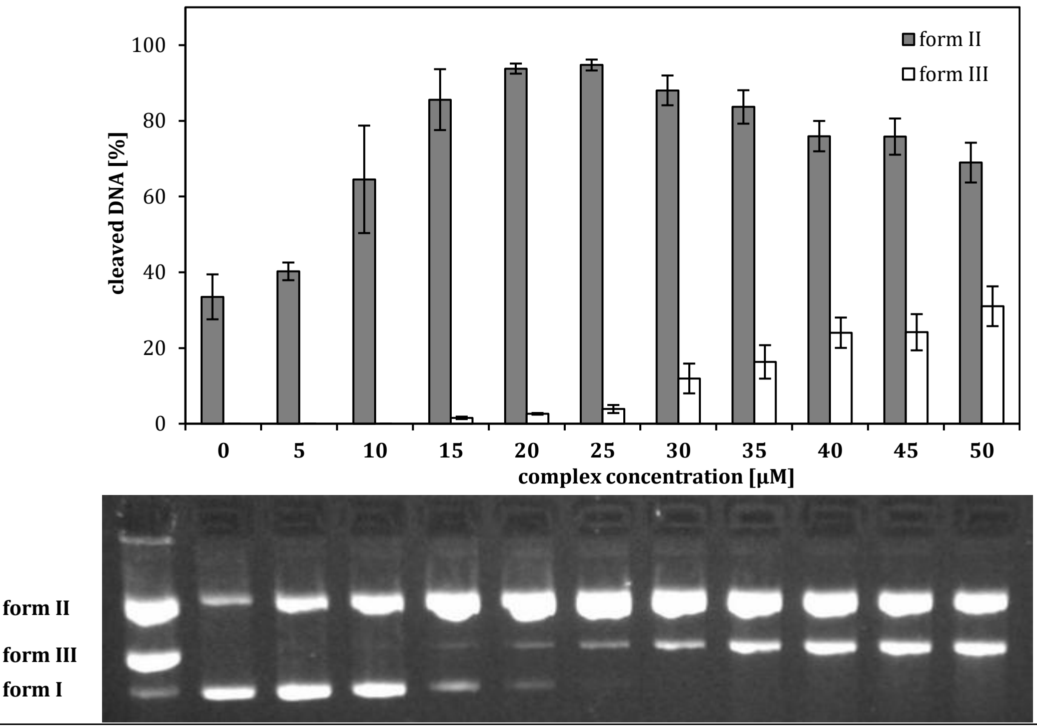


**Figure S1.1.** Cleavage activity of **CuL1** at different concentrations (in µM) towards pBR322 plasmid DNA (0.025 µg/µL) in MOPS buffer (50 mM, pH 7.4) in the presence of ascorbate (0.32 mM) for 2 h at 37 °C. Visualization of the agarose gel (**below**) and the quantitative evaluation as mean value from three measurements with the standard deviation as error bar (**above**).

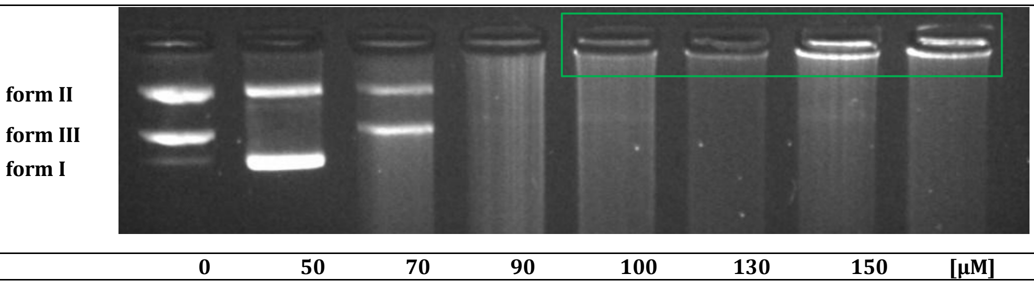


**Figure S1.2.** Cleavage activity of **CuL1** at different concentrations (in µM) towards pBR322 plasmid DNA (0.025 µg/µL) in MOPS buffer (50 mM, pH 7.4) in the presence of ascorbate (0.32 mM) for 2 h at 37 °C.

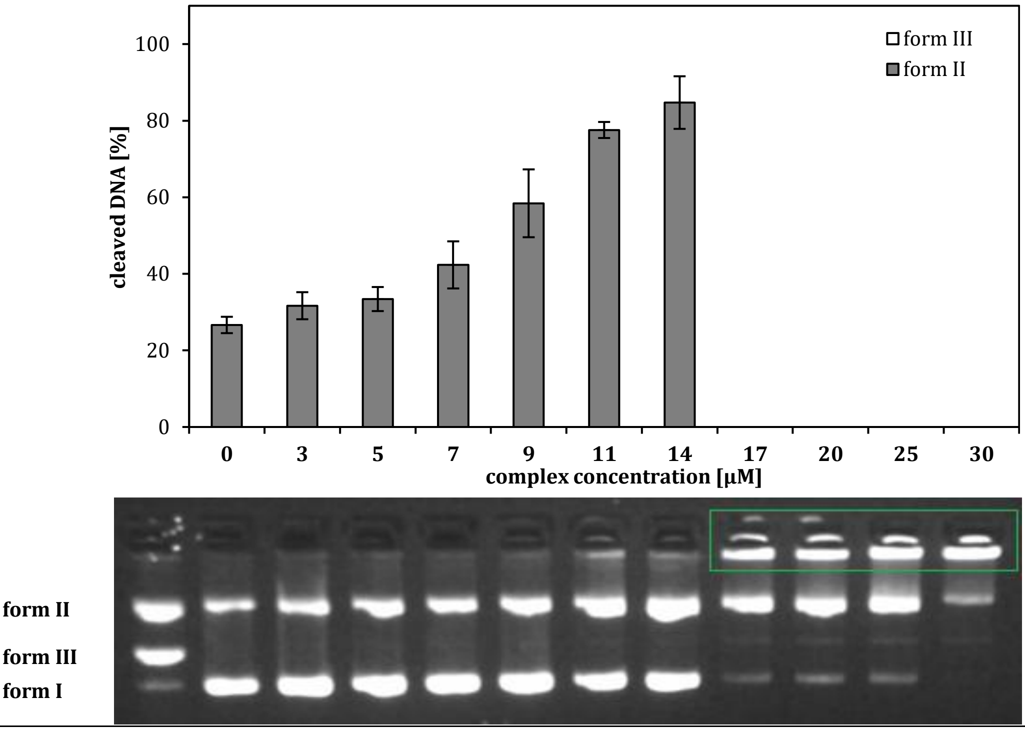


**Figure S1.3.** Cleavage activity of **CuL2** at different concentrations (in µM) towards pBR322 plasmid DNA (0.025 µg/µL) in MOPS buffer (50 mM, pH 7.4) in the presence of ascorbate (0.32 mM) for 2 h at 37 °C. Visualization of the agarose gel (**below**) and the quantitative evaluation as mean value from three measurements with the standard deviation as error bar (**above**).

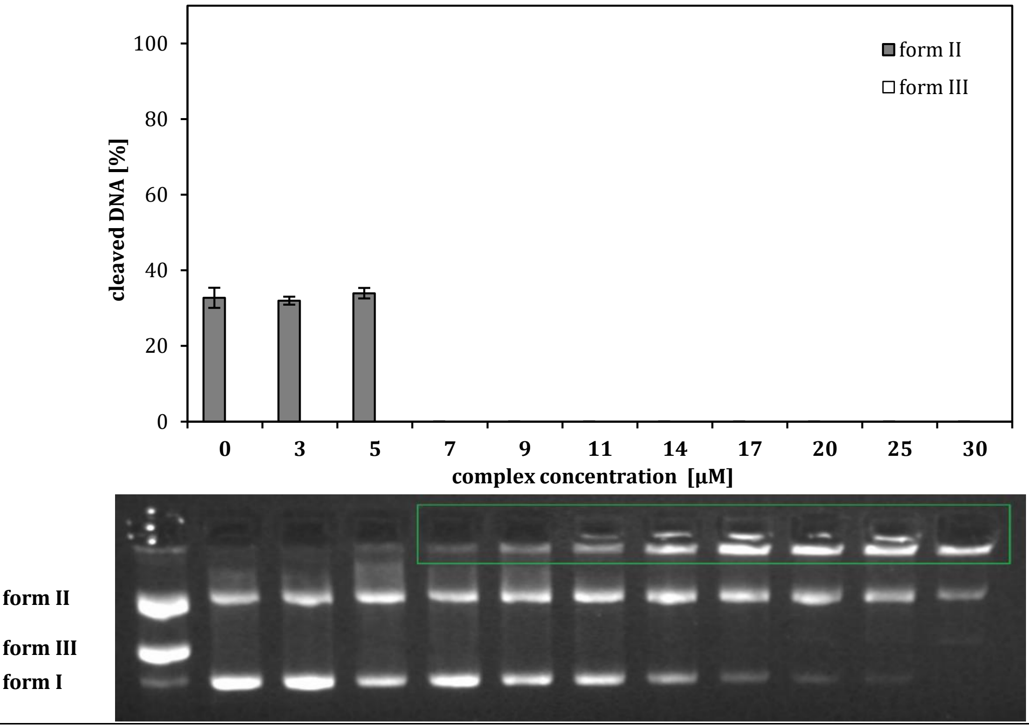


**Figure S1.4.** Cleavage activity of **CuL3** at different concentrations (in µM) towards pBR322 plasmid DNA (0.025 µg/µL) in MOPS buffer (50 mM, pH 7.4) in the presence of ascorbate (0.32 mM) for 2 h at 37 °C. Visualization of the agarose gel (**below**) and the quantitative evaluation as mean value from three measurements with the standard deviation as error bar (**above**).

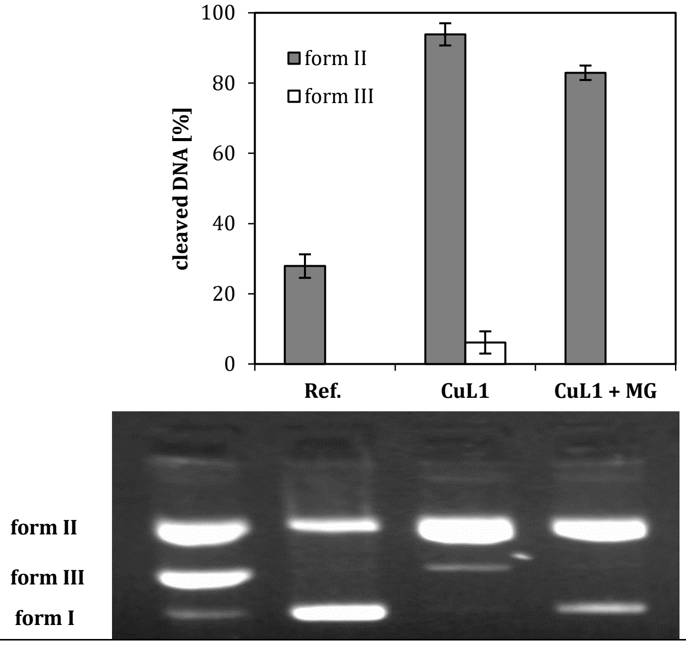


**Figure S1.5.** Cleavage activity of the complex **CuL1** (20 µM) towards pBR322 plasmid DNA (0.025 µg/µL) in MOPS buffer (50 mM, pH 7.4) and ascorbate (0.32 mM) in the presence (or absence) of DNA major groove binder methyl green (50 µM). Incubation for 2 h at 37 °C.
**Lane 1**: DNA ladder (form I, form II, form III); **lane 2**: DNA reference; **lane 3**: **CuL1** (20 µM): **lane 4**: **CuL1** + MG (50 µM). Visualization of the agarose gel (**below**). Quantitative evaluation as mean value from three measurements with the standard deviation as error bar (**above**).

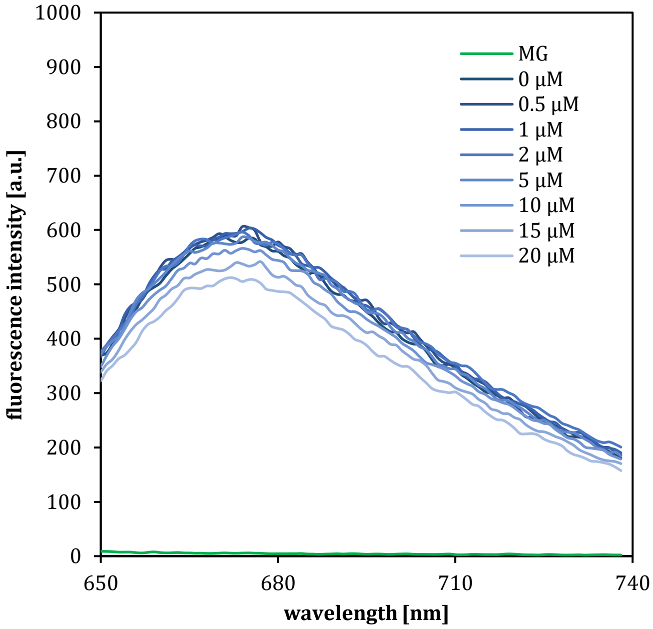


**Figure S1.6. (left)** Emission spectra of the MG-CT-DNA system (50 µM CT-DNA; 1 µM MG) in MOPS buffer (50 mM, pH 7.4) under titration of the **CuL1**.

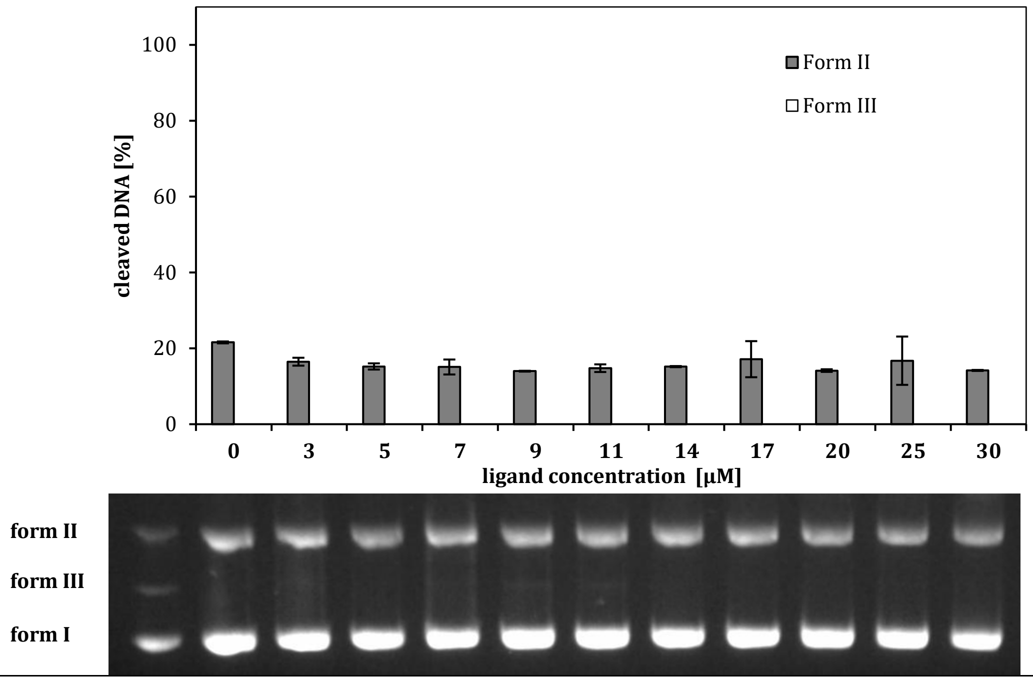


**Figure S1.7.** Cleavage activity of the ligand **L2** at different concentrations (in µM) towards pBR322 plasmid DNA (0.025 µg/µL) in MOPS buffer (50 mM, pH 7.4) in the presence of ascorbate (0.32 mM) for 2 h at 37 °C. Visualization of the agarose gel (**below**) and the quantitative evaluation as mean value from three measurements with the standard deviation as error bar (**above**).

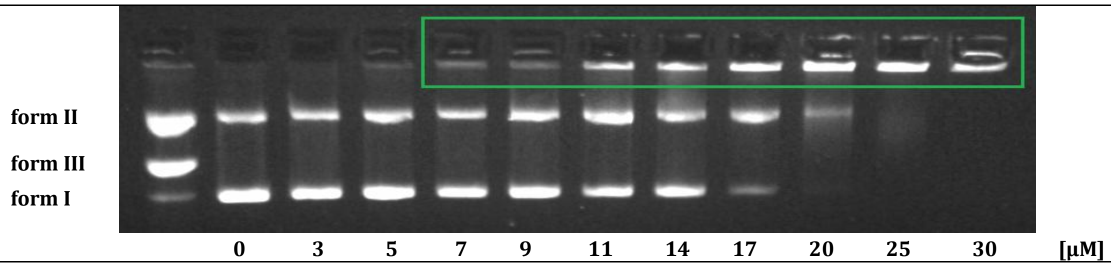


**Figure S1.8.** Cleavage activity of **CuL3** at different concentrations (in µM) towards pBR322 plasmid DNA (0.025 µg/µL) in HEPES buffer (50 mM, pH 7.4) in the presence of ascorbate (0.32 mM) for 2 h at 37 °C.

## S-2. Nuclease activity in the absence of reducing agent

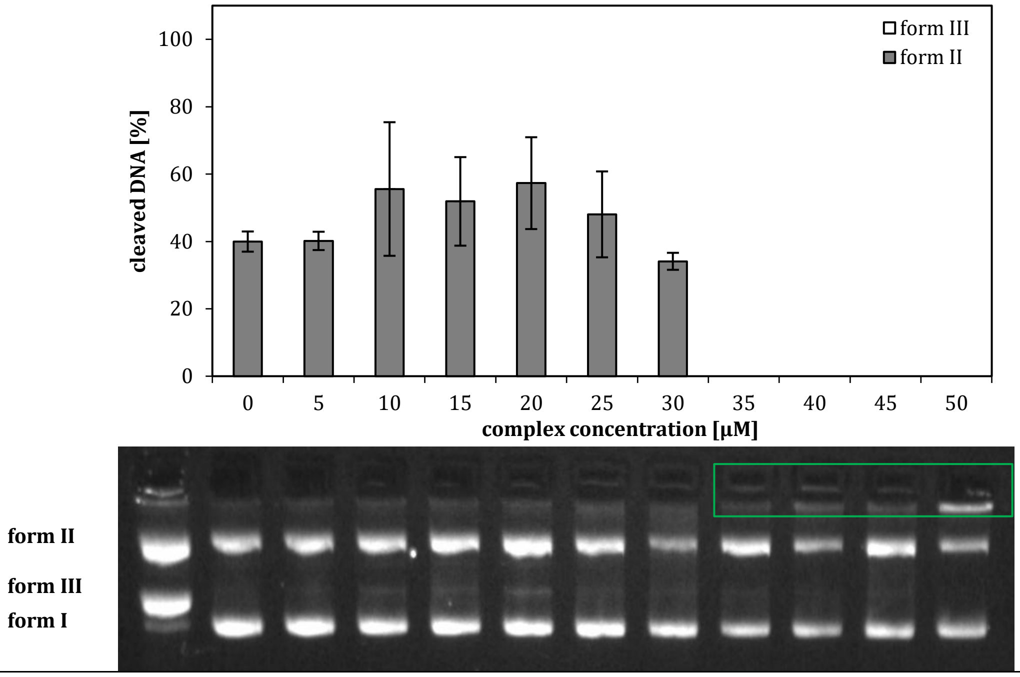


**Figure S2.1.** Cleavage activity of **CuL1** at different concentrations (in µM) towards pBR322 plasmid DNA (0.025 µg/µL) in MOPS buffer (50 mM, pH 7.4) for 24 h at 37 °C. Visualization of the agarose gel (**below**) and the quantitative evaluation as mean value from three measurements with the standard deviation as error bar (**above**).

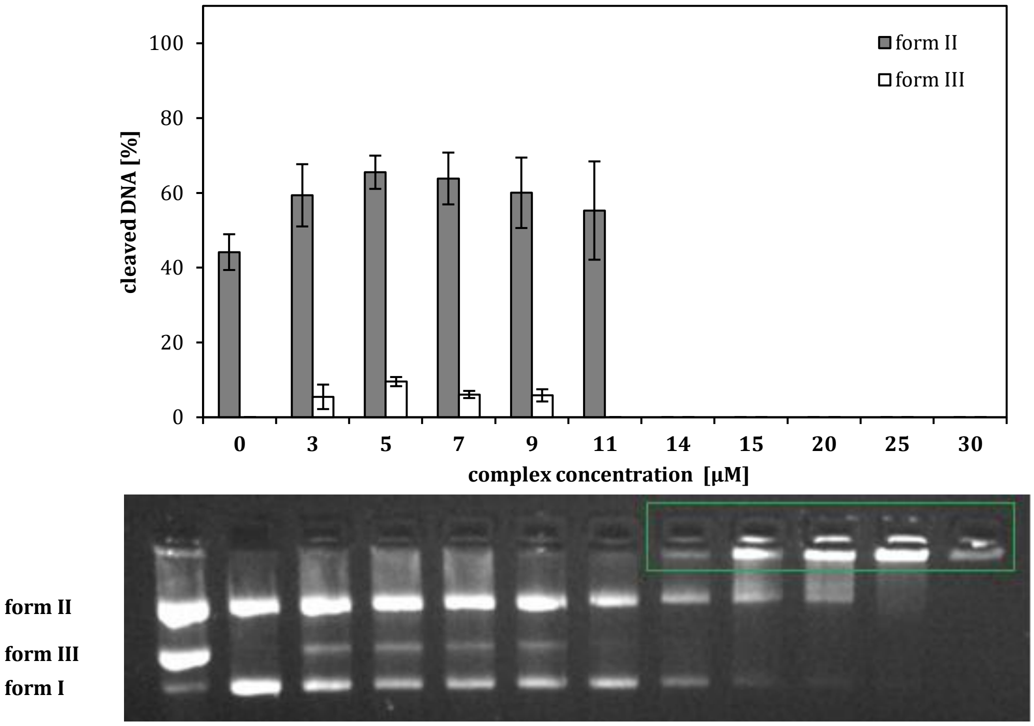


**Figure S2.2.** Cleavage activity of **CuL2** at different concentrations (in µM) towards pBR322 plasmid DNA (0.025 µg/µL) in MOPS buffer (50 mM, pH 7.4) for 24 h at 37 °C. Visualization of the agarose gel (**below**) and the quantitative evaluation as mean value from three measurements with the standard deviation as error bar (**above**).

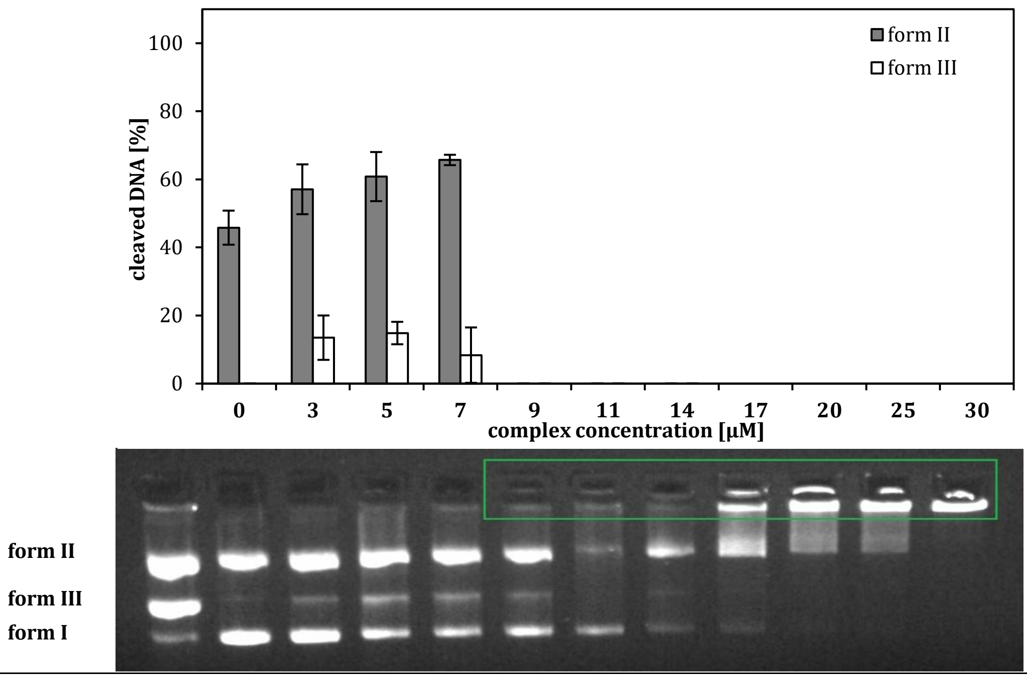


**Figure S2.3.** Cleavage activity of **CuL3** at different concentrations (in µM) towards pBR322 plasmid DNA (0.025 µg/µL) in MOPS buffer (50 mM, pH 7.4) for 24 h at 37 °C. Visualization of the agarose gel (**below**) and the quantitative evaluation as mean value from three measurements with the standard deviation as error bar (**above**).

## S-3. Bis(4-nitrophenyl) phosphate assay

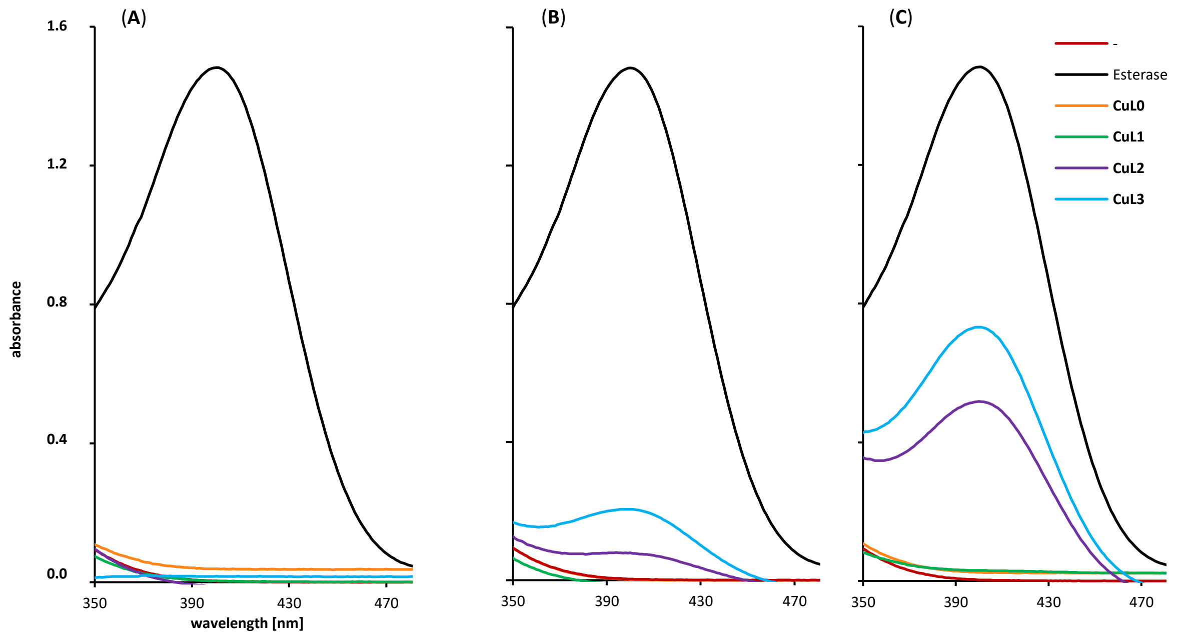


**Figure S3.** Absorption spectra of BNPP (0.08 mM) in MOPS buffer (50 mM, pH 7,4) in the presence of phosphodiesterase I enzyme (0.05 U/mL) or **CuL0** – **CuL3** (0.2 mM) after 0 (**A**), 2 (**B**) and 24 h (**C**) incubation at 37 °C.

## S-4. Detection of hydroxyl radicals and hydrogen peroxide

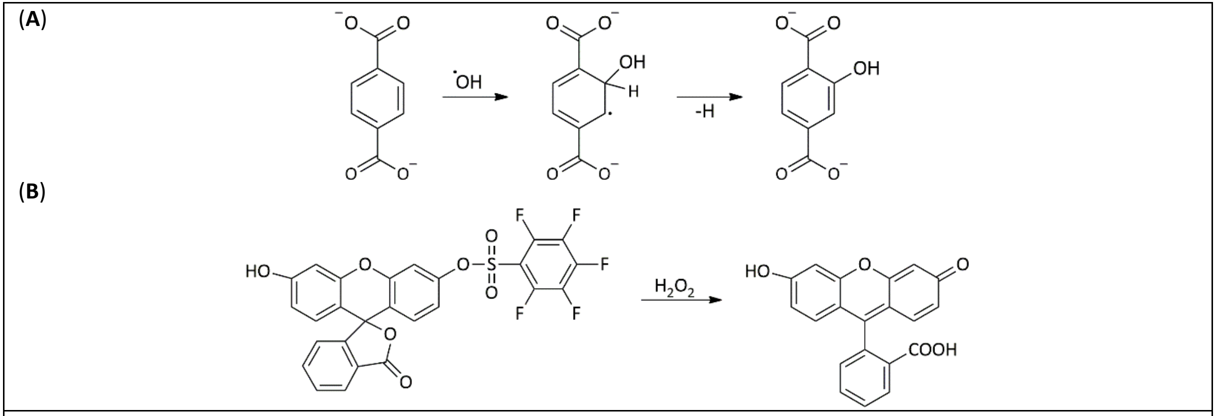


**Figure S4.1.** (**A**) Oxidation of the terepthalate (TPA) to the highly fluorescent 2-hydroxyterepthalate (HTPA) by hydroxyl radical. (**B**) Perhydrolysis of the pentafluorobenzenesulfonyl fluorescein (PBSF) to fluorescein.

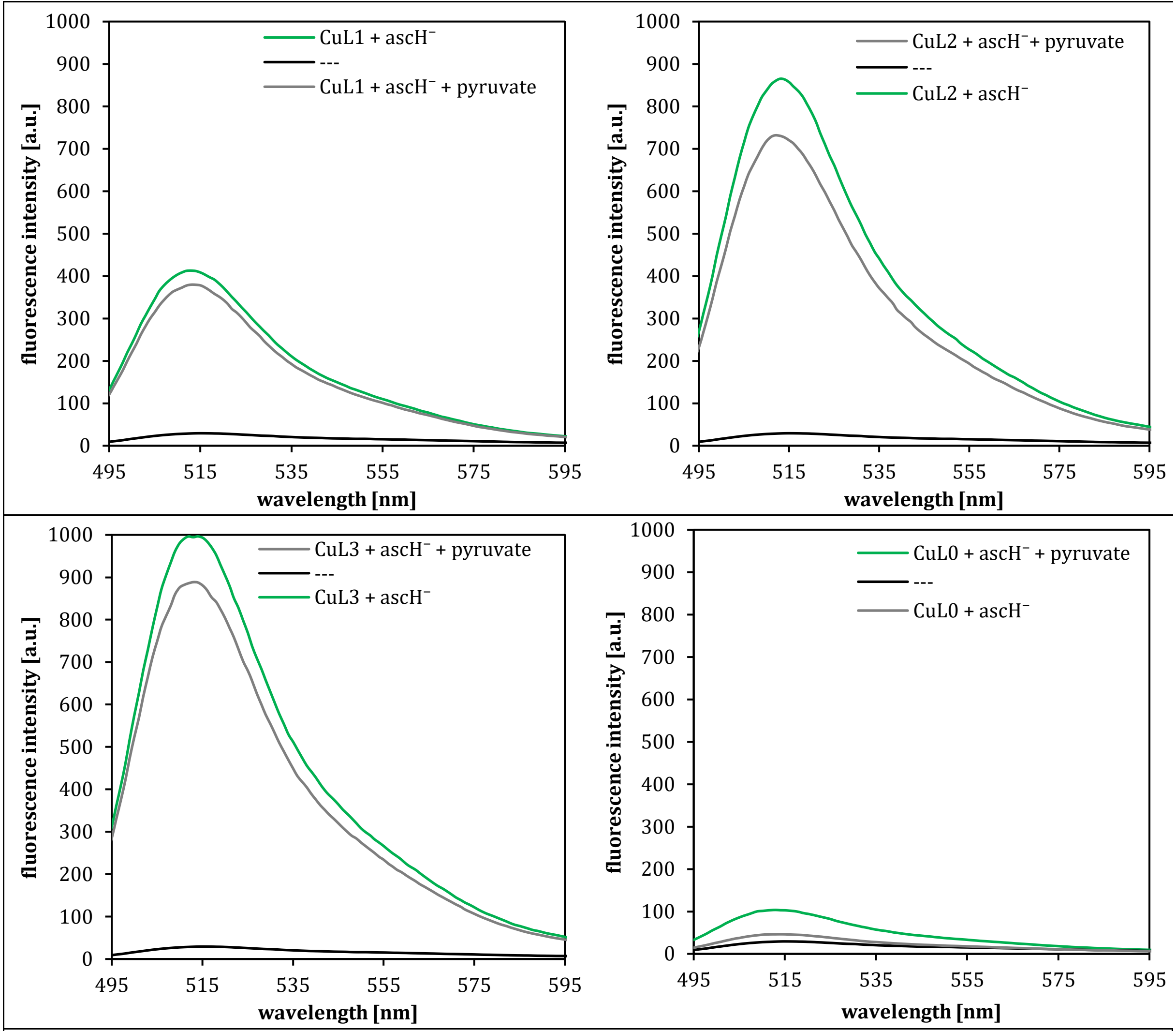


**Figure S4.2.** Emission spectra of PBSF (25 µM) in the presence of the complex **CuL1** (40 µM) (left, above) / **CuL2** (40 µM) (right, above) / **CuL3** (40 µM) (left, below) / **CuL0** (40 µM) (right, below), ascorbic acid (1 mM), pyruvate (10 mM) in MOPS buffer (50 mM, pH 7.4). Incubation for 2.5 h at 37 °C.

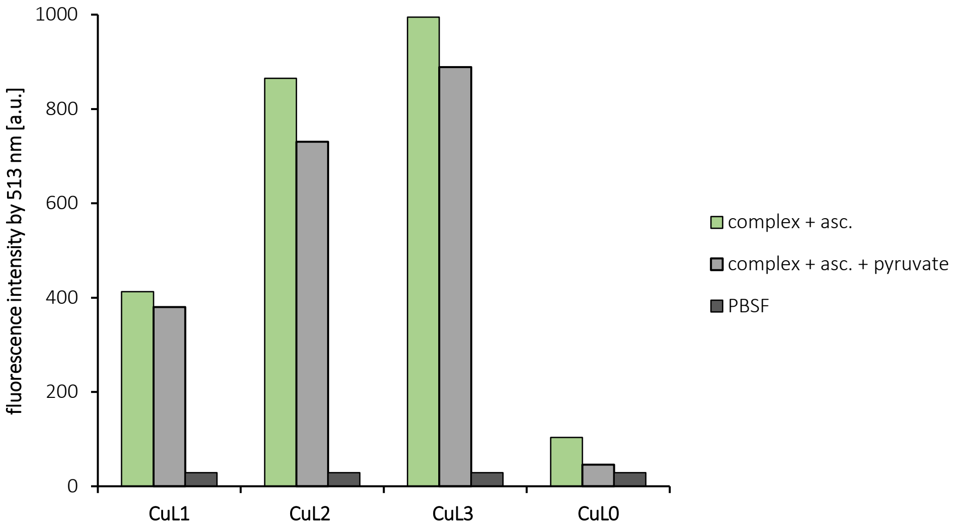


**Figure S4.3.** Fluorescence emission maxima for PSBF (0.25 mM) showing the intensity obtained for $H_2O_2$ production **CuL0**, **CuL1**, **CuL2** and **CuL3** (40 µM) in the presence of ascorbic acid (1 mM) in MOPS buffer (50 mM, pH 7.4) and scavenging agents pyruvate (10 mM) ($\lambda_{ex}$ 485 nm, $\lambda_{em}$ = 513 nm).

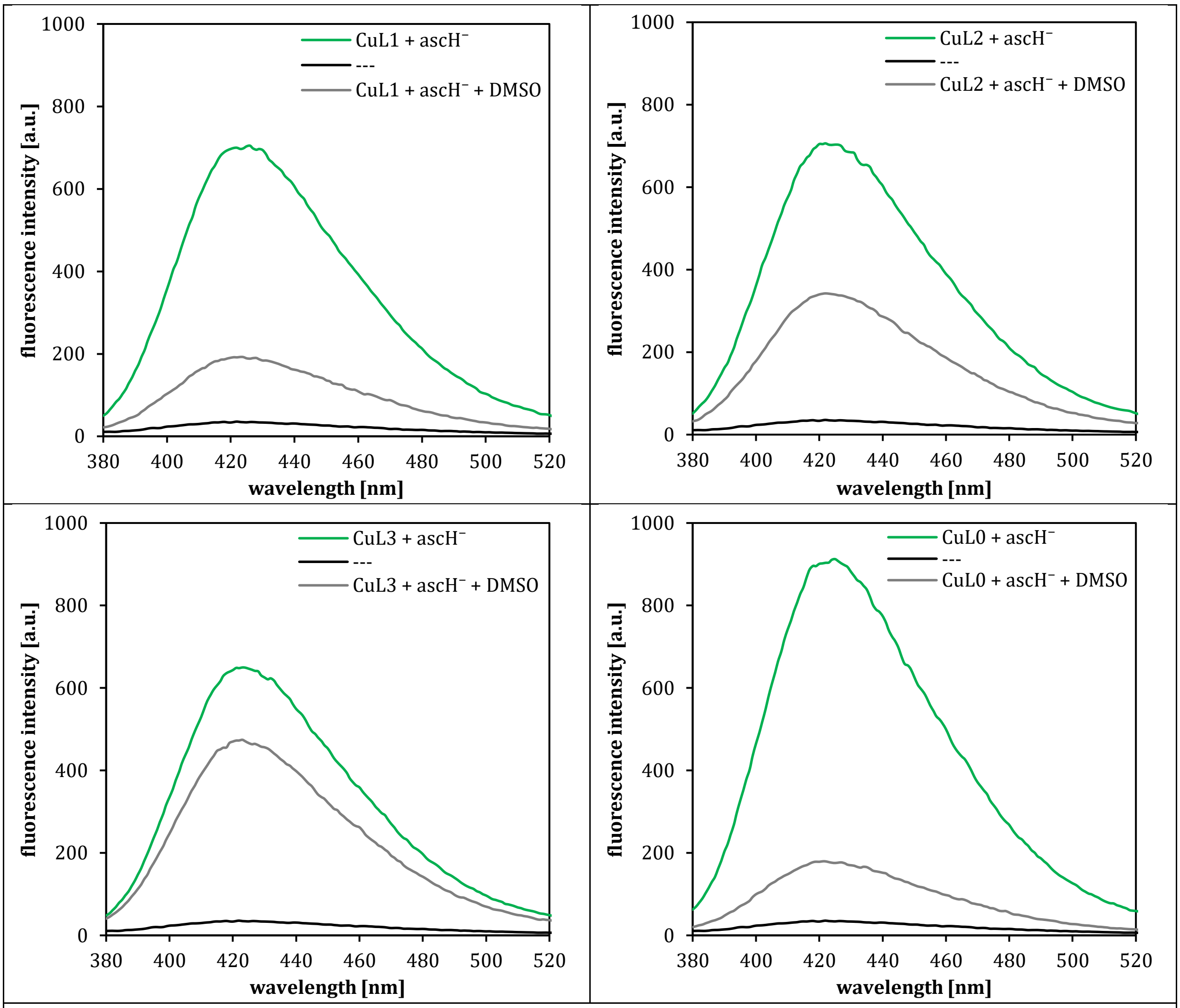


**Figure S4.4.** Emission spectra of the TPA (50 µM) in the presence of the complex **CuL1** (40 µM) (left, top) / **CuL2** (40 µM) (right, top) / **CuL3** (40 µM) (left, bottom) / **CuL0** (40 µM) (right, bottom), ascorbic acid (0.25 µM), DMSO (200 mM) in MOPS buffer (50 mM, pH 7.4). Incubation for 2.5 h at 37 °C.

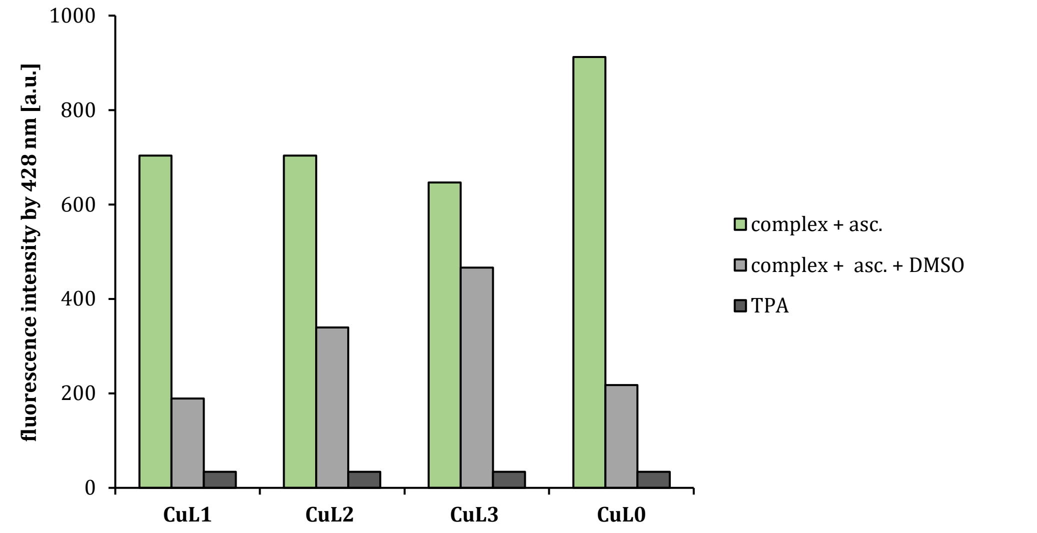


**Figure S4.5.** Fluorescence emission maxima for TPA (0.5 mM) showing the intensity obtained for ·OH production **CuL0**, **CuL1**, **CuL2** and **CuL3** (40 µM) in the presence of ascorbic acid (1 mM) in MOPS buffer (50 mM, pH 7.4) and scavenging agents DMSO (200 mM) ($\lambda_{ex}$ 320 nm, $\lambda_{em}$ = 428 nm).

## S-5. Ethidium bromide displacement assay

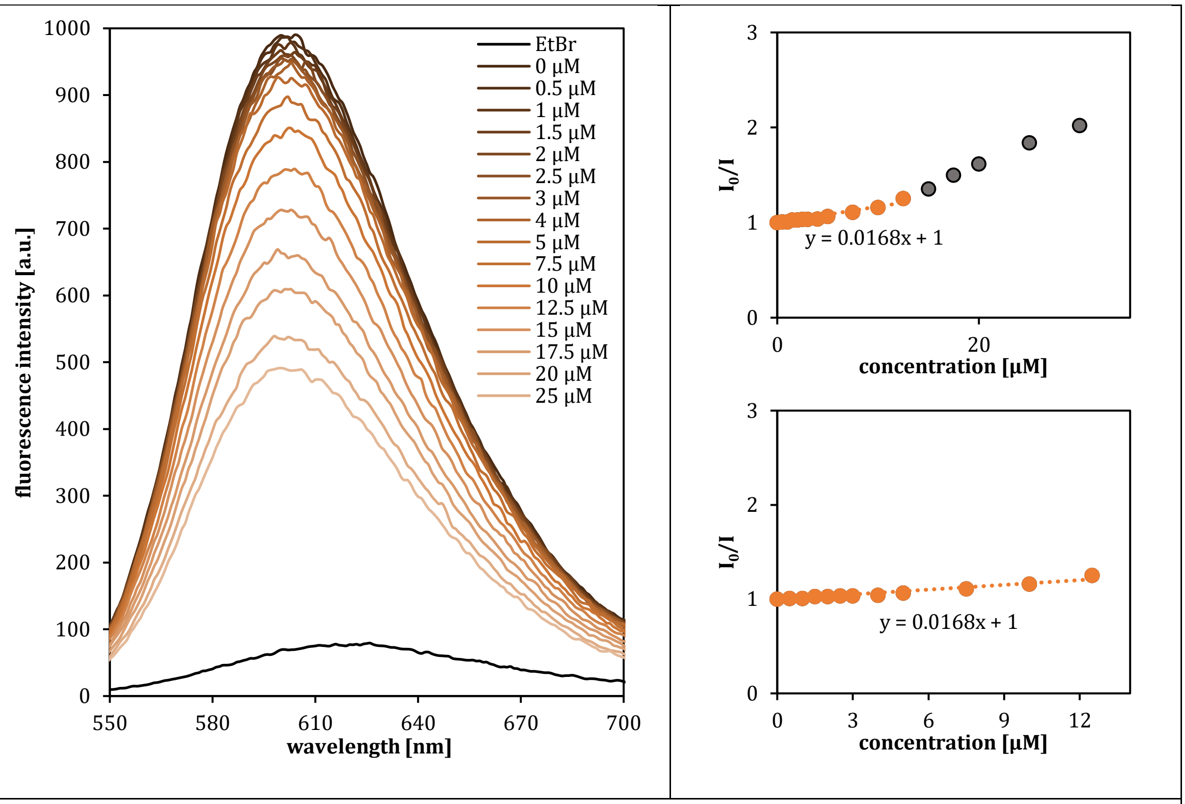


**Figure S5.1. (left)** Emission spectra of the EtBr-CT-DNA system (20 µM CT-DNA; 1.3 µM EtBr) in MOPS buffer (50 mM, pH 7.4) under titration of **$Cu(NO_3)_2$. (right)** Graphical representation of the $I_0/I$ over [$Q$] for the determination of $K_{SV}$ and $K_{app}$.

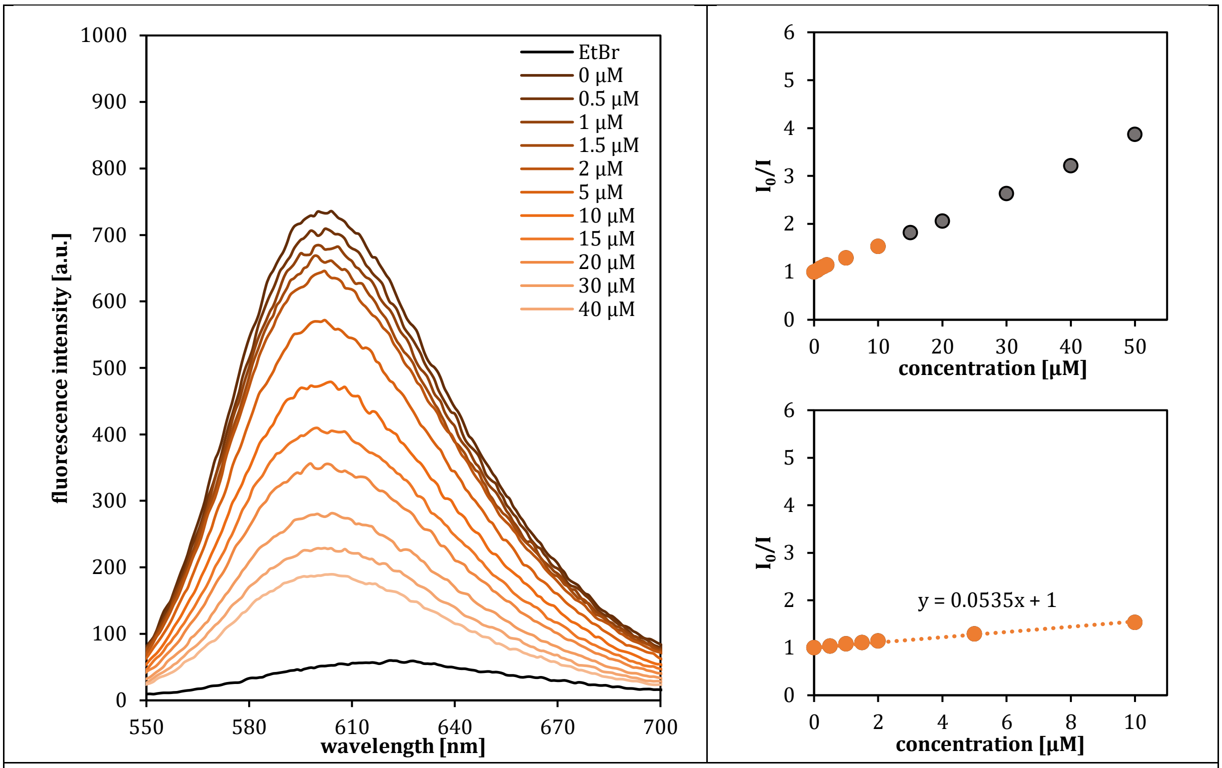


**Figure S5.2. (left)** Emission spectra of the EtBr-CT-DNA system (20 µM CT-DNA; 1.3 µM EtBr) in MOPS buffer (50 mM, pH 7.4) under titration of **CuL0**. **(right)** Graphical representation of the $I_0/I$ over $[Q]$ for the determination of $K_{SV}$ and $K_{app}$.

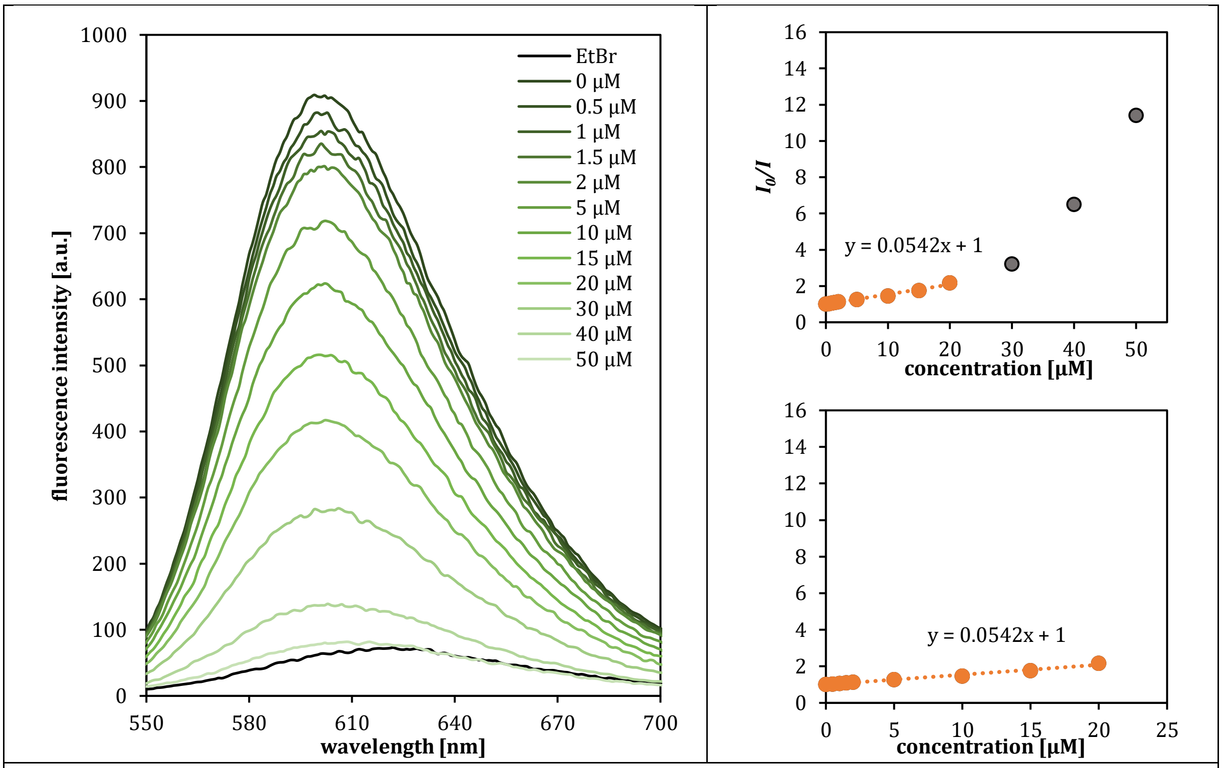


**Figure S5.3. (left)** Emission spectra of EtBr-CT-DNA system (20 µM CT-DNA; 1.3 µM EtBr) in MOPS buffer (50 mM, pH 7.4) under titration of **CuL1**. **(right)** Graphical representation of the $I_0/I$ over $[Q]$ for the determination of $K_{SV}$ and $K_{app}$.

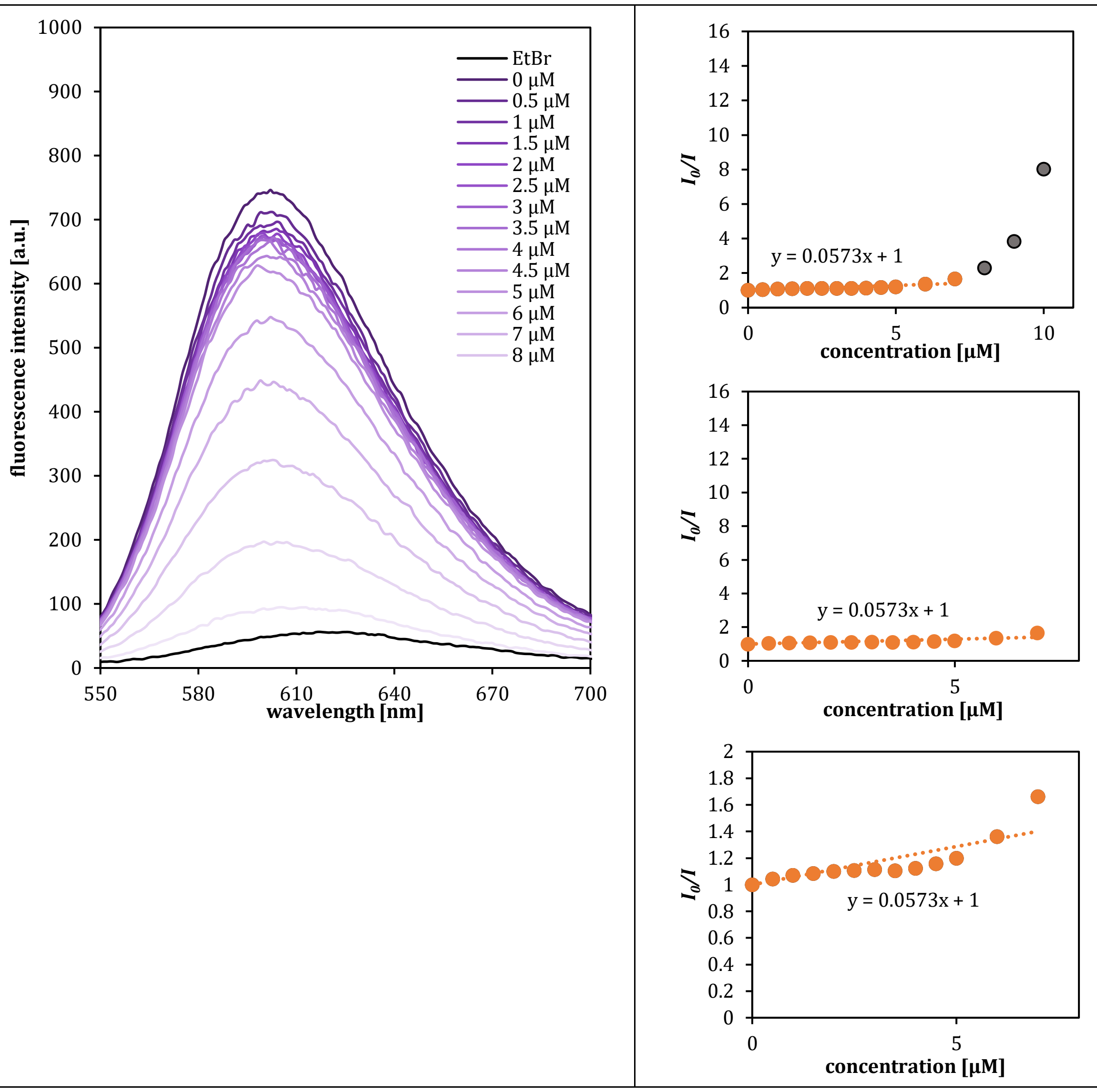


| |
|---|
| **Figure S5.4. (left)** Emission spectra of the EtBr-CT-DNA system (20 µM CT-DNA; 1.3 µM EtBr) in MOPS buffer (50 mM, pH 7.4) under titration of the **CuL2**. **(right)** Graphical representation of the $I_0/I$ over [$Q$] for the determination of $K_{SV}$ and $K_{app}$. |

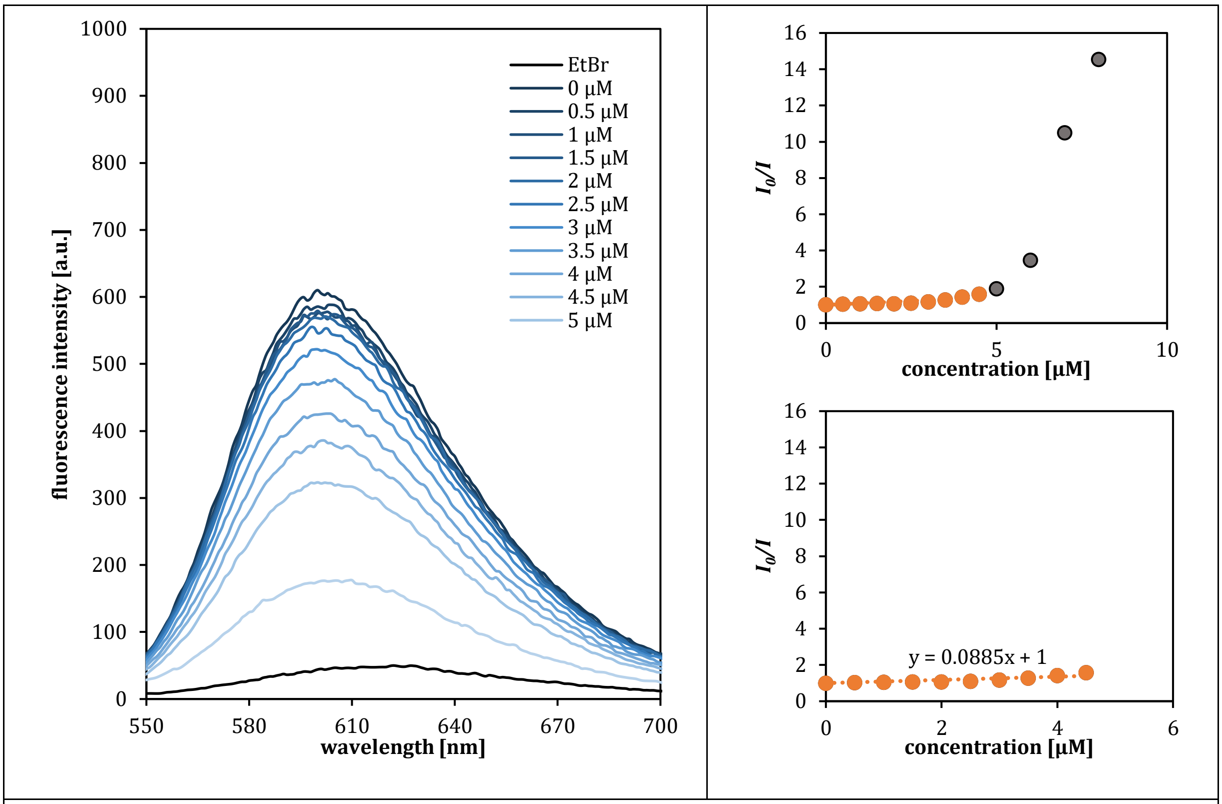


**Figure S5.5. (left)** Emission spectra of the EtBr-CT-DNA system (20 µM CT-DNA; 1.3 µM EtBr) in MOPS buffer (50 mM, pH 7.4) under titration of **CuL3**. **(right)** Graphical representation of the $I_0/I$ over $[Q]$ for the determination of $K_{SV}$ and $K_{app}$.

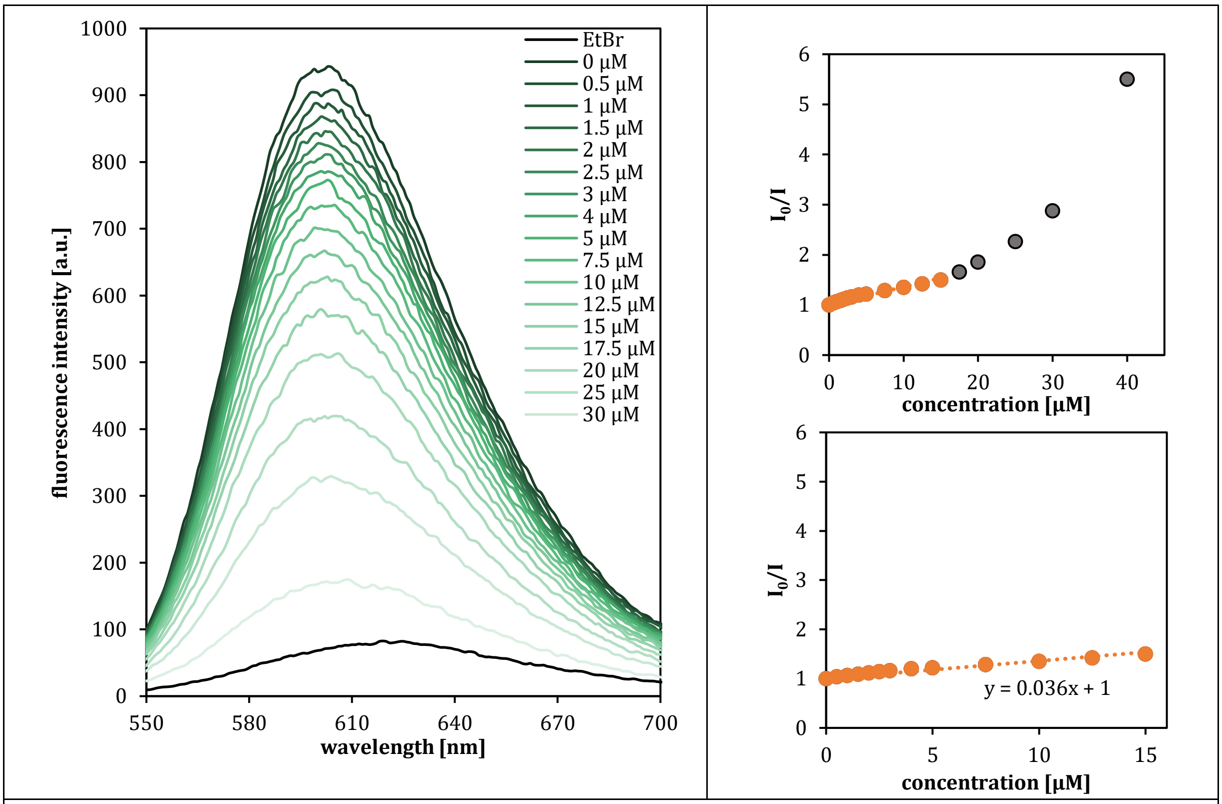


**Figure S5.6. (left)** Emission spectra of the EtBr-CT-DNA system (20 µM CT-DNA; 1.3 µM EtBr) in MOPS buffer (50 mM, pH 7.4) under titration of the ligand of the complex **CuL1**. **(right)** Graphical representation of the $I_0/I$ over $[Q]$ for the determination of $K_{SV}$ and $K_{app}$.

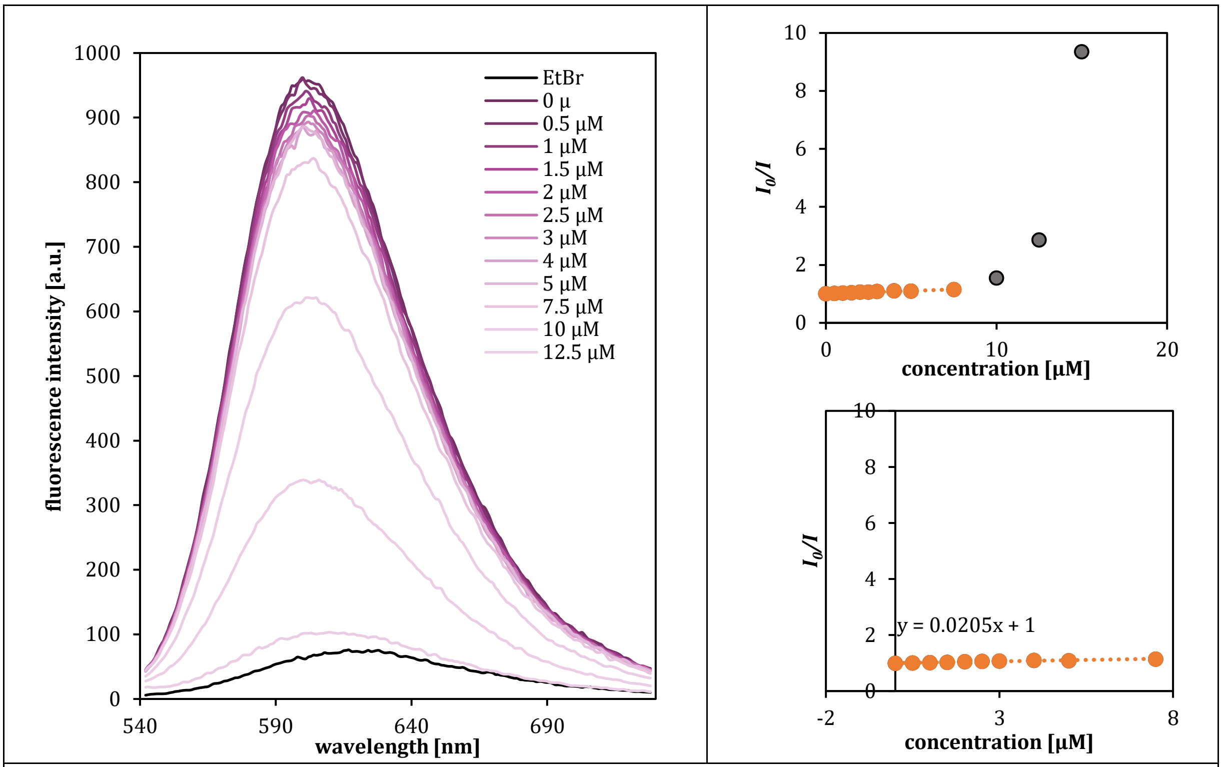


**Figure S5.7. (left)** Emission spectra of the EtBr-CT-DNA system (20 µM CT-DNA; 1.3 µM EtBr) in MOPS buffer (50 mM, pH 7.4) under titration of the ligand of the complex **CuL2**. **(right)** Graphical representation of the $I_0/I$ over [$Q$] for the determination of $K_{SV}$ and $K_{app}$.

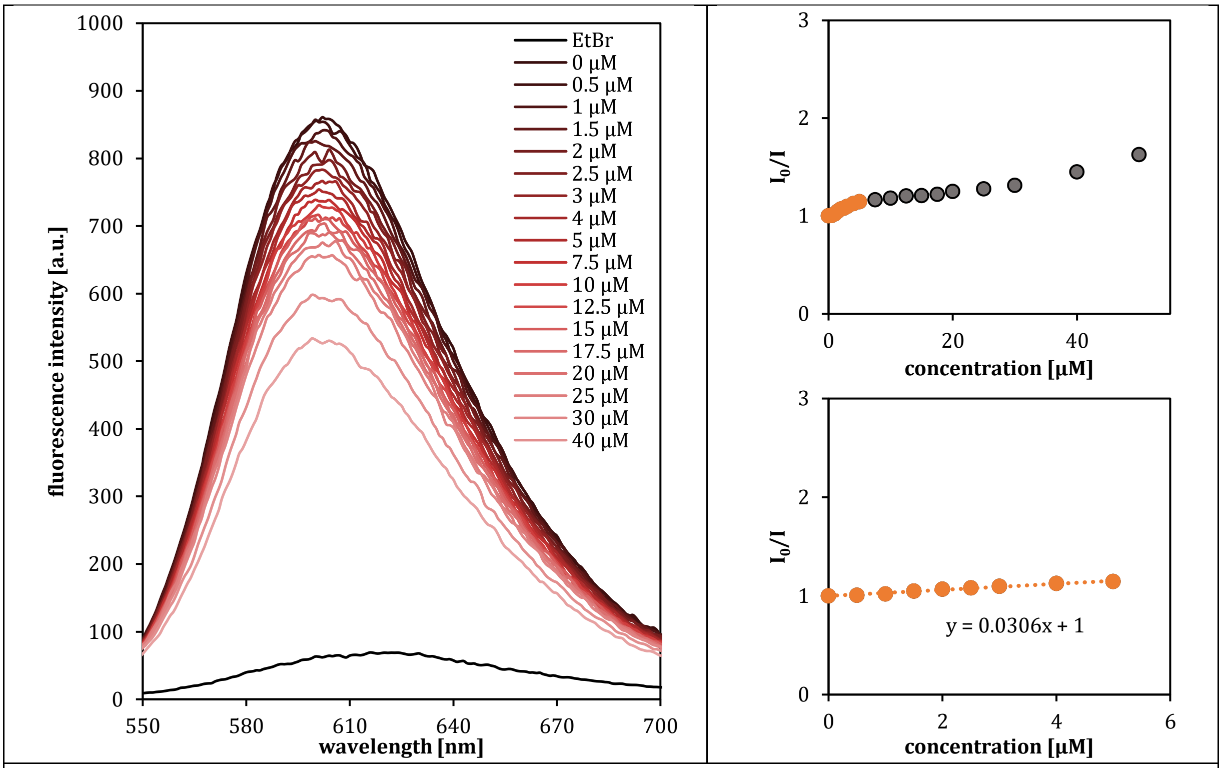


**Figure S5.8. (left)** Emission spectra of the EtBr-CT-DNA system (20 µM CT-DNA; 1.3 µM EtBr) in MOPS buffer (50 mM, pH 7.4) under titration of the ligand of the complex **CuL3**. **(right)** Graphical representation of the $I_0/I$ over [$Q$] for the determination of $K_{SV}$ and $K_{app}$.

## S-6. Circular dichroism spectroscopy

(1) **CuL0**

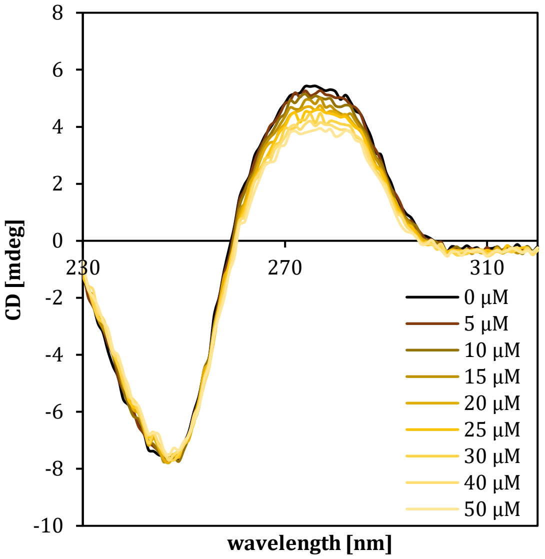


(2) **CuL1**

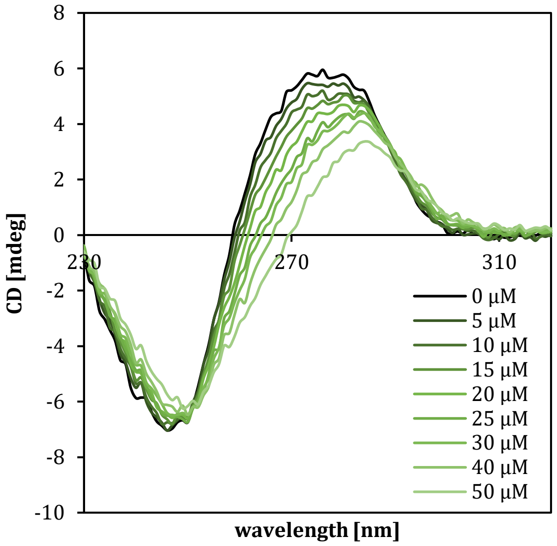


(3) **CuL2**

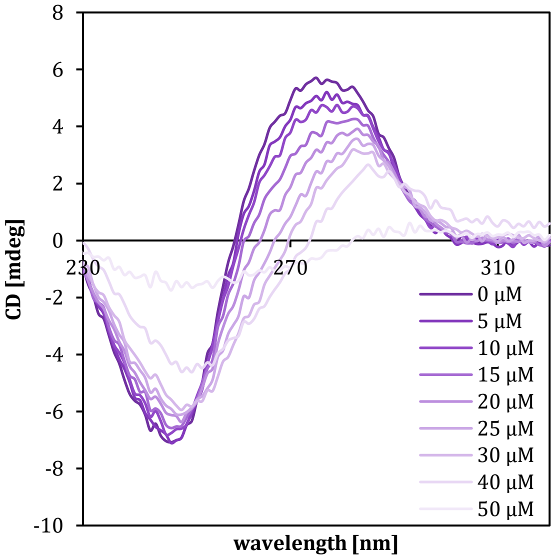


(4) **CuL3**

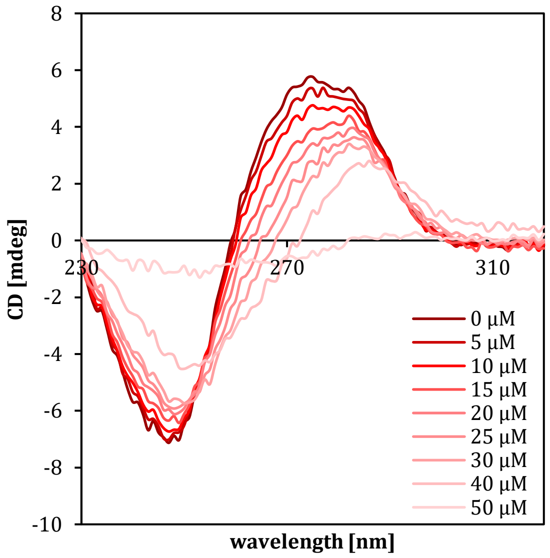


**Figure S6.1.** Circular dichroism of the CT-DNA (100 µM) in MOPS (50 mM, pH7.4) with increasing concentration of the Cu(II) complexes **CuL0–CuL3** (0–50 µM).

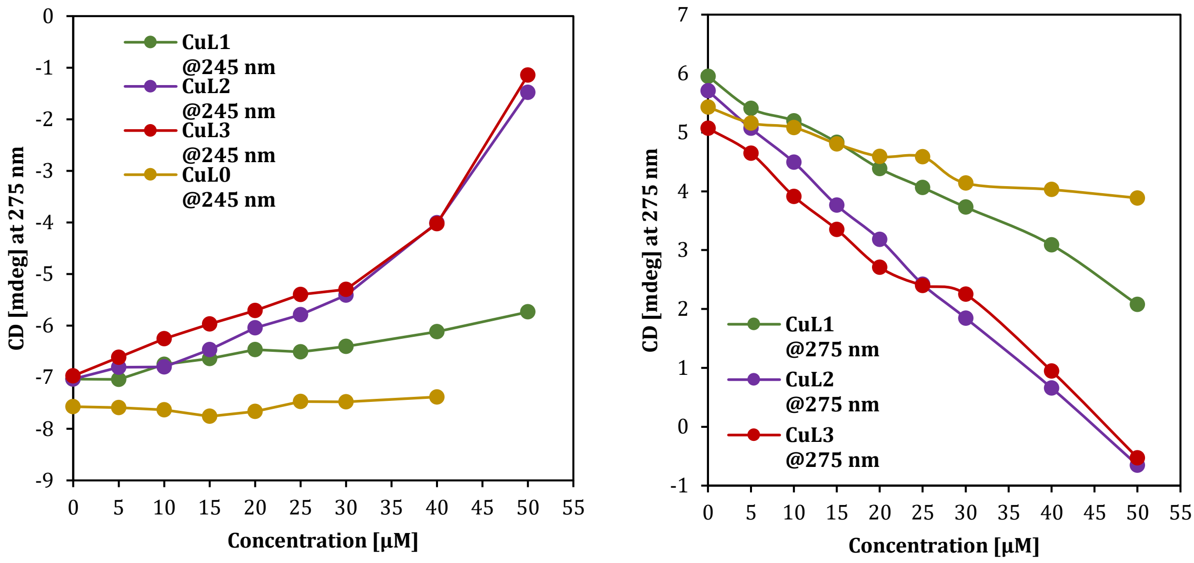


**Figure S6.2.** Circular dichroism intensities at 245 nm (**left**) and 275 nm (**right**) of CT-DNA (100 µM) in MOPS (50 mM, pH7.4) the Cu(II) complexes **CuL0–CuL3** (0–50 µM).

## S-7. DNA melting point determination

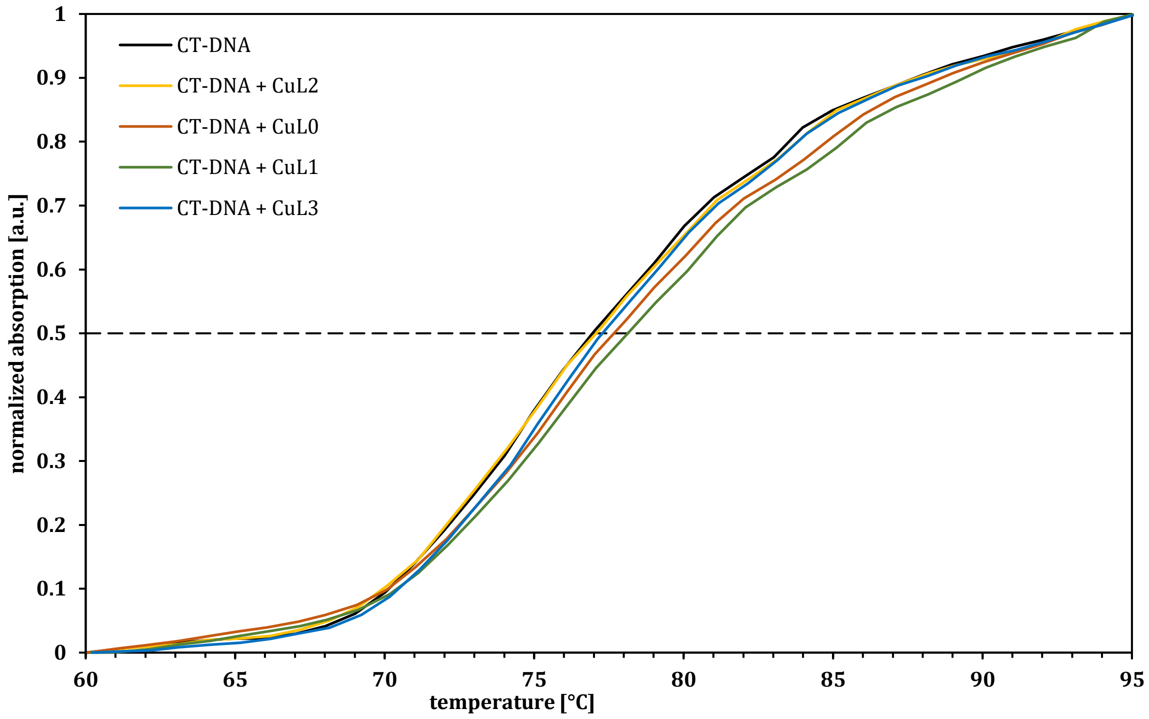


**Figure S7.** Graphical representation of the normalized melting point temperature ($T_m$) of CT-DNA (100 µM) in MOPS buffer (50 mM, pH 7.4) in the absence (reference) and in the presence of **CuL0**, **CuL1**, **CuL2** and **CuL3** at 260 nm.

| | $T_m$ [°C] | $\Delta T_m$ |
|---|---|---|
| **CT-DNA** | 76.99 ± 0.03 | – |
| **+CuL0** | 78.10 ± 0.10 | + 1.12 (± 0.13) |
| **+CuL1** | 78.12 ± 0.10 | + 1.13 (± 0.13) |
| **+CuL2** | 77.10 ± 0.06 | + 0.12 (± 0.15) |
| **+CuL3** | 77.47 ± 0.52 | + 0.48 (± 0.55) |

**Table S7.:** Melting point temperature $T_m$ of Cu(II) complexes **CuL0 – CuL3** and the temperature difference $\Delta T_m$.

## S-8. Atomic force microscopy

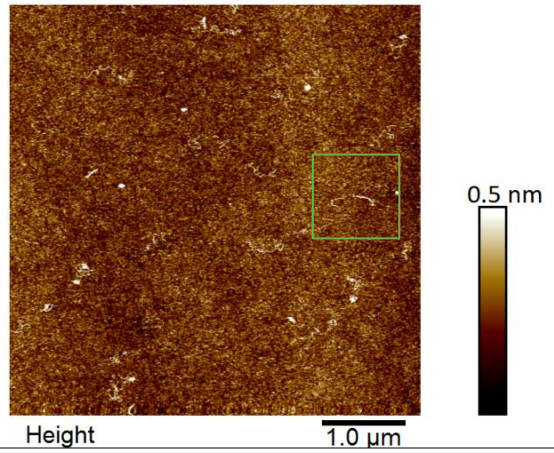


**Figure S8.1.** Representative AFM image of linearized pBR322 plasmid DNA (20 ng) before addition of the Cu(II) complexes.

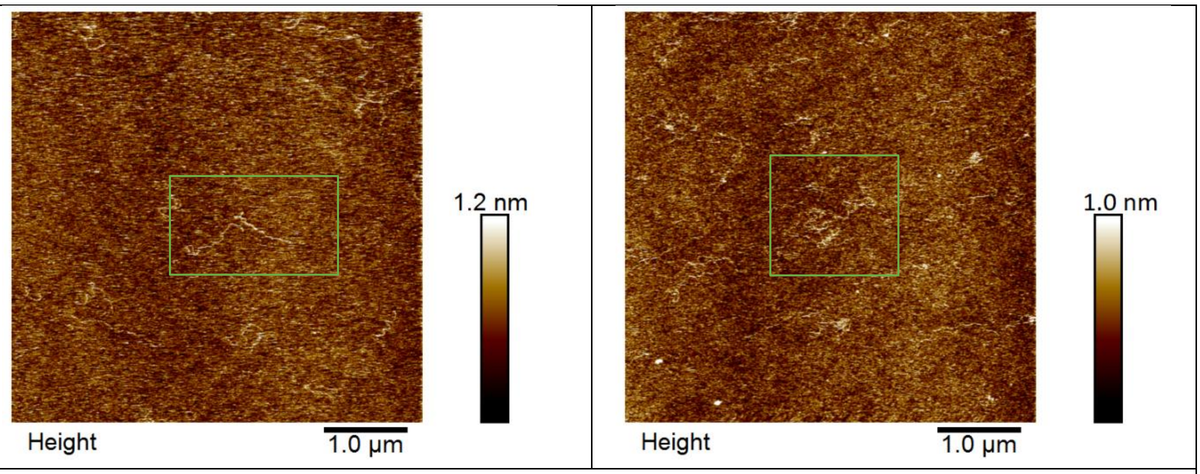


**Figure S8.2.** Representative AFM image of linearized pBR322 plasmid DNA (20 ng) in the presence of **CuL1** at 3 µM (**left**) and 30 µM (**right**).

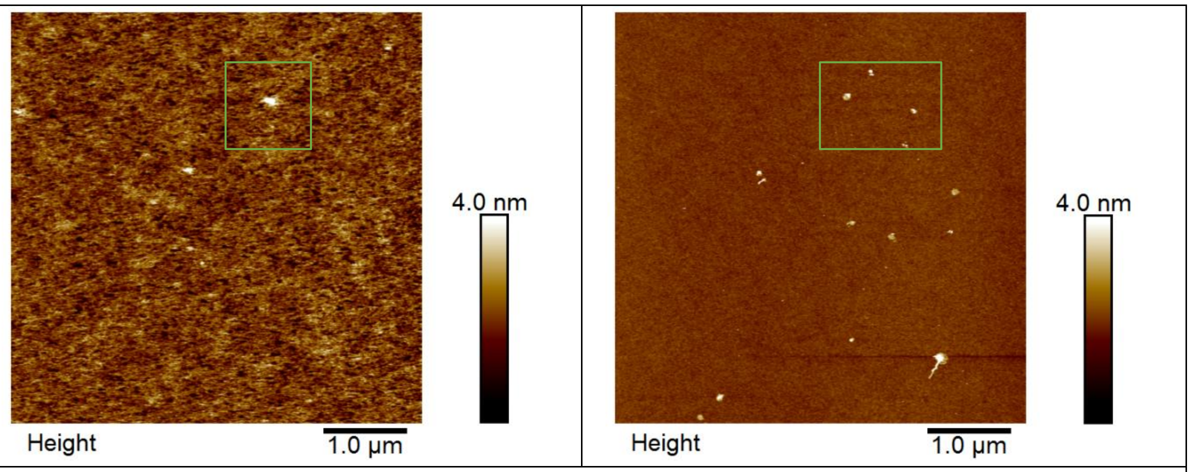


**Figure S8.3.** Representative AFM image of linearized pBR322 plasmid DNA (20 ng) in the presence of **CuL2** at 3 µM (**left**) and 30 µM (**right**).

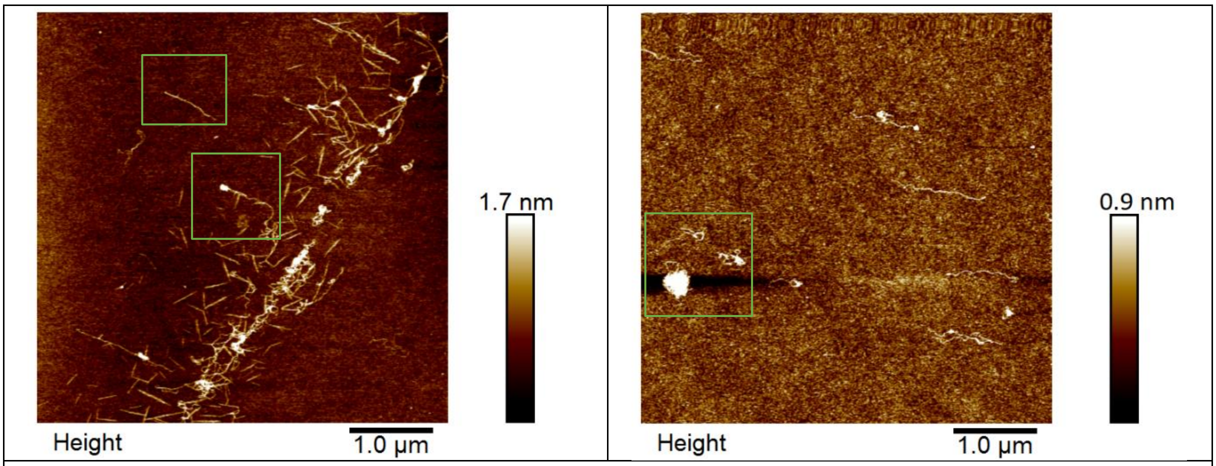


**Figure S8.4.** Representative AFM image of linearized pBR322 plasmid DNA (20 ng) in the presence of **CuL3** at 3 µM (**left**) and 30 µM (**right**).

## S-9. Dynamic light scattering

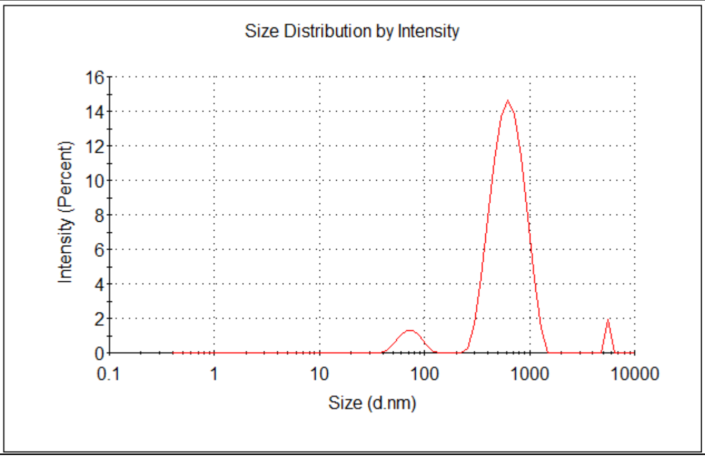


**Figure S9.1.** Size distribution by intensity of 30 µM of **CuL1** in MOPS buffer (50 mM, pH 7.4) and 0.025 µg/µL pBR322 plasmid DNA at 37 °C after incubation time of 2 min before DLS measurement.

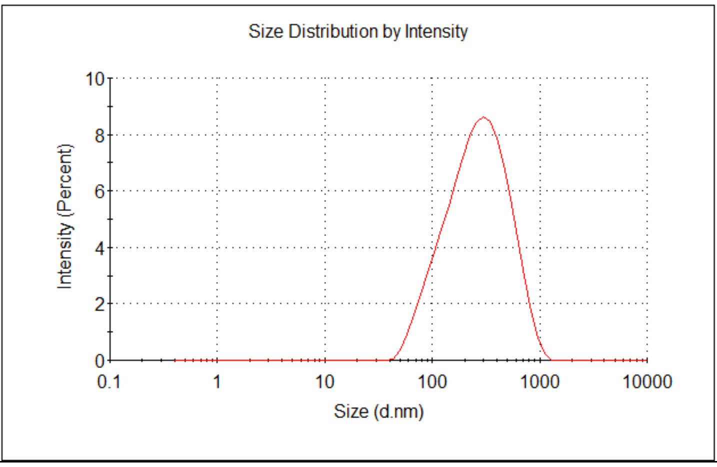


**Figure S9.2.** Size distribution by intensity of 30 µM of **CuL2** in MOPS buffer (50 mM, pH 7.4) and 0.025 µg/µL pBR322 plasmid DNA at 37 °C after incubation time of 2 min before DLS measurement.

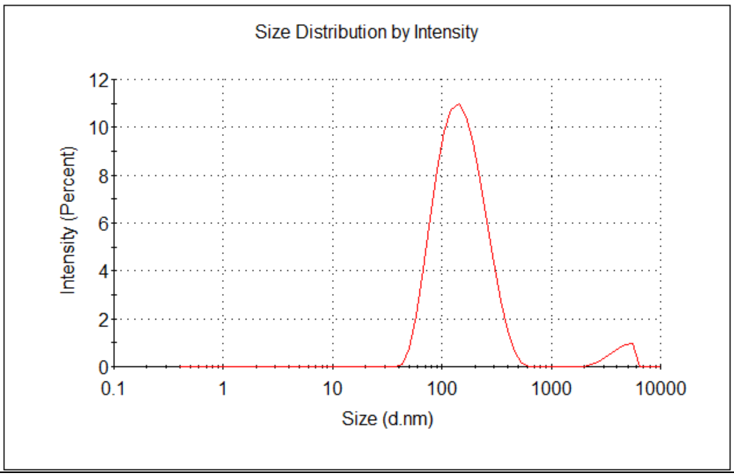


**Figure S9.3.** Size distribution by intensity of 30 µM of **CuL3** in MOPS buffer (50 mM, pH 7.4) and 0.025 µg/µL pBR322 plasmid DNA at 37 °C after incubation time of 2 min before DLS measurement.
